# Comparative Genomics Uncovers the Evolutionary Dynamics of Detoxification and Insecticide Target Genes Across 11 Phlebotomine Sand Flies

**DOI:** 10.1093/gbe/evae186

**Published:** 2024-09-03

**Authors:** Jason Charamis, Sofia Balaska, Panagiotis Ioannidis, Vít Dvořák, Konstantinos Mavridis, Mary Ann McDowell, Pavlos Pavlidis, René Feyereisen, Petr Volf, John Vontas

**Affiliations:** Department of Biology, University of Crete, Heraklion 71409, Greece; Institute of Molecular Biology and Biotechnology, Foundation for Research and Technology-Hellas, Heraklion 70013, Greece; Department of Biology, University of Crete, Heraklion 71409, Greece; Institute of Molecular Biology and Biotechnology, Foundation for Research and Technology-Hellas, Heraklion 70013, Greece; Institute of Molecular Biology and Biotechnology, Foundation for Research and Technology-Hellas, Heraklion 70013, Greece; Institute of Computer Science, Foundation for Research and Technology-Hellas, Heraklion, Greece; Department of Parasitology, Faculty of Science, Charles University, Prague, Czech Republic; Institute of Molecular Biology and Biotechnology, Foundation for Research and Technology-Hellas, Heraklion 70013, Greece; Eck Institute for Global Health, Department of Biological Sciences, University of Notre Dame, Notre Dame, IN, USA; Department of Biology, University of Crete, Heraklion 71409, Greece; Institute of Computer Science, Foundation for Research and Technology-Hellas, Heraklion, Greece; Laboratory of Agrozoology, Department of Plants and Crops, Faculty of Bioscience Engineering, Ghent University, Ghent 9000, Belgium; Department of Parasitology, Faculty of Science, Charles University, Prague, Czech Republic; Institute of Molecular Biology and Biotechnology, Foundation for Research and Technology-Hellas, Heraklion 70013, Greece; Pesticide Science Laboratory, Department of Crop Science, Agricultural University of Athens, Athens 11855, Greece

**Keywords:** phlebotomine sand flies, comparative genomics, gene family evolution, cytochrome P450s, xenobiotic adaptation

## Abstract

Sand flies infect more than 1 million people annually with *Leishmania* parasites and other bacterial and viral pathogens. Progress in understanding sand fly adaptations to xenobiotics has been hampered by the limited availability of genomic resources. To address this gap, we sequenced, assembled, and annotated the transcriptomes of 11 phlebotomine sand fly species. Subsequently, we leveraged these genomic resources to generate novel evolutionary insights pertaining to their adaptations to xenobiotics, including those contributing to insecticide resistance. Specifically, we annotated over 2,700 sand fly detoxification genes and conducted large-scale phylogenetic comparisons to uncover the evolutionary dynamics of the five major detoxification gene families: cytochrome P450s (CYPs), glutathione-S-transferases (GSTs), UDP-glycosyltransferases (UGTs), carboxyl/cholinesterases (CCEs), and ATP-binding cassette (ABC) transporters. Using this comparative approach, we show that sand flies have evolved diverse CYP and GST gene repertoires, with notable lineage-specific expansions in gene groups evolutionarily related to known xenobiotic metabolizers. Furthermore, we show that sand flies have conserved orthologs of (i) CYP4G genes involved in cuticular hydrocarbon biosynthesis, (ii) ABCB genes involved in xenobiotic toxicity, and (iii) two primary insecticide targets, acetylcholinesterase-1 (Ace1) and voltage gated sodium channel (VGSC). The biological insights and genomic resources produced in this study provide a foundation for generating and testing hypotheses regarding the molecular mechanisms underlying sand fly adaptations to xenobiotics.

**Significance**

The diversity and evolution of gene families underlying the physiological adaptations of sand flies to xenobiotic compounds remain poorly understood, largely due to the limited availability of genomic resources. Here we generate transcriptome assemblies for 11 phlebotomine sand fly species and leverage them to elucidate the evolutionary trajectories of the five major detoxification gene families in these arthropod vectors. In addition to providing crucially missing genomic resources, our data significantly advance our understanding on how xenobiotic detoxification has evolved in sand flies. Importantly, we show that most of the cytochrome P450 and glutathione-S-transferase diversity is caused by multiple lineage-specific expansions of gene groups evolutionarily related to known xenobiotic metabolizers, suggesting candidate genes for future molecular studies on sand fly adaptations to xenobiotics.

## Introduction

Phlebotomine sand flies are major arthropod vectors of various human and animal pathogens, including *Leishmania* parasites, as well as other pathogens of bacterial or viral origin. While mostly occurring in tropical and subtropical climates, in recent decades, ongoing climatic and environmental changes favor their expansion to new and previously non-endemic geographical regions. This trend is expected to intensify in the coming years as suggested by models of environmental and climatic suitability as well as empirical field data (Medlock et al. 2014). The emergence of competent sand fly vectors will be reflected by a change in the epidemiology of sand fly-borne diseases. Recent increases in autochthonous leishmaniasis cases in previously non-endemic regions, including areas north of the traditional sand fly distribution, have already been documented in Europe (Maia et al. 2023).

As sand fly-borne diseases are regarded as neglected and the sand fly terrestrial life cycle with loosely defined breeding sites is not easily targeted, sand fly control is often achieved indirectly by insecticide-based interventions against mosquitoes (Wilson et al. 2020). Such non-targeted approaches typically fail to effectively suppress sand fly populations, highlighting the need to develop efficient chemical control strategies that are specifically designed against sand flies (Balaska et al. 2021). Furthermore, these strategies should explicitly consider insecticide resistance, as the malaria paradigm demonstrates that the benefits of chemical-based control can be quickly lost due to the development of resistance (Wilson et al. 2020). The occurrence of insecticide resistance in sand flies may seem not to be widespread when compared to malaria mosquito vectors, but this picture may be biased as available data are sparsely distributed and often inconsistently sourced (Balaska et al. 2021). Several cases of confirmed or suspected resistance to pyrethroids and DDT have been recently accumulating in India, Sudan and Iran where systematic control interventions and data collection exist (Balaska et al. 2021).

Insecticide resistance is caused by toxicokinetic and toxicodynamic changes in insect physiology (Feyereisen et al. 2015). Increased metabolism and/or excretion of insecticides is the most prevalent toxicokinetic change leading to resistance (Feyereisen et al. 2015). Insects have developed sophisticated mechanisms to detoxify xenobiotics, including insecticides, toxic plant allelochemicals, and environmental pollutants (Cruse et al. 2023). Detoxification capacity largely depends on five gene/enzyme superfamilies with distinct catalytic functions: cytochrome P450 monooxygenases (CYPs), glutathione-S-transferases (GSTs), uridine diphosphate-glycosyltransferases (UGTs), and carboxyl/cholinesterases (CCEs), implicated in the metabolism/sequestration of toxicants, and ATP-binding cassette (ABC) transporters, responsible for excreting the detoxified products (Cruse et al. 2023). The toxicokinetic changes causing resistance predominantly result from cis and/or trans upregulation of genes in those superfamilies, as well as their copy number variation (CNV). We currently lack critical knowledge about the evolutionary trajectories and divergence patterns of these five detoxification superfamilies in sand flies. Gaining insights into these processes will be of great significance in our effort to elucidate the underlying molecular mechanisms of sand fly adaptations against xenobiotics, including those contributing to insecticide resistance. The most relevant toxicodynamic change leading to insecticide resistance is reduced sensitivity of insecticide targets mainly caused by nonsynonymous mutations affecting highly conserved protein domains, which are important for their physiological function. Changes in target site gene expression or CNV have been observed and may compensate for the fitness deficit caused by the point mutations (Feyereisen et al. 2015).

Progress in sand fly research has been hampered by the limited availability of genomic resources. Recently, the genomes of two important vectors of human leishmaniases, namely *Phlebotomus papatasi* and *Lutzomyia longipalpis*, were sequenced and analyzed (Labbé et al. 2023). However, this only accounts for a fraction of species of epidemiological relevance: approximately 100 species of the currently described 1,000 species are regarded as proven or suspected vectors incriminated in various human-infecting *Leishmania* species in the Old and New World (Maroli et al. 2013). Genomic resources are not available for representative species of these lineages, limiting our ability to perform comparative evolutionary and molecular studies pertaining to their adaptations to xenobiotics. Thus, we sequenced and assembled the whole body transcriptomes of 11 sand fly species which originate from Africa, Asia, Europe, and Latin America. With a focus on species closely related to the two major human leishmaniases vectors, *P. papatasi* and *L. longipalpis*, the sampled sand flies include proven vectors of visceral and cutaneous leishmaniasis, and span three main traditionally recognized Phlebotominae genera, *Phlebotomus*, *Lutzomyia*, and *Sergentomyia*, which shared a common ancestor more than 200 million years ago (Akhoundi et al. 2016).

Taking advantage of the multiple species comparisons, we explored the evolutionary dynamics of the five main detoxification gene families (CYPs, GSTs, UGTs, CCEs, ABC transporters) in phlebotomines and analyzed the sequence of a primary insecticide target. Our data significantly advance our understanding on how xenobiotic detoxification has evolved in phlebotomines and provide fundamental genomic resources, which will facilitate future studies on the molecular mechanisms underpinning sand fly adaptations to natural and synthetic xenobiotics.

## Materials and Methods

### Sand Fly Rearing

Specimens analyzed in this study were reared under standardized conditions as described by Volf and Volfova (2011) in the insectary of the Department of Parasitology, Faculty of Science, Charles University, Prague. Specimens of 11 species belonging to three traditionally recognized genera and eight subgenera were analyzed (country of origin and year of colony establishment in Prague is given in brackets): *Lutzomyia* (*Lutzomyia*) *longipalpis* (Brazil, 1991), *L.* (*Migonemyia*) *migonei* (Brazil, 2014), *P.* (*Adlerius*) *arabicus* (Israel, 2001), *P.* (*Euphlebotomus*) *argentipes* (India, 2008), *P.* (*Phlebotomus*) *duboscqi* (Senegal, 2008), *P.* (*Larroussius*) *orientalis* (Ethiopia, 2010), *P.* (*Phlebotomus*) *papatasi* (Turkey, 2005), *P.* (*Larroussius*) *perniciosus* (Spain, 1994), *P.* (*Paraphlebotomus*) *sergenti* (Turkey, 2011), *P.* (*Larroussius*) *tobbi* (Turkey, 2008), and *Sergentomyia* (*Sergentomyia*) *schwetzi* (Portugal, 2020).

### RNA Isolation and Library Preparation

Whole body RNA was extracted from 3- to 5-d-old female, non-blood fed sand flies, using TRIzol reagent (Thermo Fisher Scientific) and, subsequently, treated with DNase (Invitrogen TURBO DNA-free kit, Ambion), both used according to the manufacturer's instructions. For each sand fly species, either two or three biological replicates, each containing seven to eight individuals, were prepared. The quantity and quality of the isolated RNA were estimated with a NanoDrop ND-1000 Spectrophotometer and 2100 Agilent Bioanalyzer, respectively.

RNA samples (1 μg of each) were shipped to Macrogen Europe BV (Amsterdam, The Netherlands) for mRNA library construction using the Illumina TruSeq Stranded mRNA sample preparation kit and sequenced in the Illumina NovaSeq platform yielding 150 bp paired-end reads (supplementary table S1, Supplementary Material online).

### Transcriptome Assembly

We produced transcriptome assemblies using Trinity v2.15.0 (Grabherr et al. 2011) and rnaSPAdes v3.15.5 (Bushmanova et al. 2019), which were recently recognized among the transcriptome assemblers with consistently good performance across multiple datasets (Hölzer and Marz 2019). Subsequently, we combined the Trinity- and rnaSPAdes-produced assemblies using EvidentialGene (Gilbert 2013) with default parameters (supplementary fig. S1, Supplementary Material online). The 11 sand fly transcriptome assemblies are provided in the Supplementary Material (supplementary file S1, Supplementary Material online).

### Gene Prediction

We performed gene prediction in the EvidentialGene-produced transcriptome assemblies using TransDecoder v5.5.0 with integration of HMMer 3.3.2 searches for conserved Pfam domains (Eddy 2011) and DIAMOND 2.0.15 (Buchfink et al. 2021) blastp searches for conserved proteins from the SwissProt database (Coudert et al. 2023). Last, we also filtered out identical and nearly identical unigenes at the protein level (≥99% local sequence identity) using CD-HIT 4.8.1 (Fu et al. 2012). The complete transcriptome assembly and gene prediction pipeline is described in supplementary fig. S1, Supplementary Material online and provided as an automated Snakemake workflow (https://github.com/JasonCharamis/SandFlyComparativeGenomics/blob/main/pipelines/01.transcriptome_assembly_and_gene_prediction/workflow/Snakefile). The 11 sand fly gene sets are provided in the Supplementary Material (supplementary file S2, Supplementary Material online).

### Quality Assessment of Transcriptome Assemblies and Gene Sets

We evaluated transcriptome assemblies using TransRate v1.0.3 (Smith-Unna et al. 2016) and BUSCO v5.1.2 (Manni et al. 2021). TransRate evaluates de novo transcriptome assemblies by mapping the reads back to the assembled transcripts and generating various quality metrics derived from this mapping (Smith-Unna et al. 2016). BUSCO evaluates the quality of genomic data based on the presence of universal single-copy orthologs (Manni et al. 2021). Assessment using both approaches demonstrated that Trinity consistently produced assemblies with higher completeness but less accuracy compared to rnaSPAdes (supplementary tables S2 and S3, Supplementary Material online). Evigene assemblies had consistenly equally high completeness and much lower redundancy compared to both Trinity and rnaSPAdes assemblies (supplementary tables S3 and S4, Supplementary Material online). Sand fly gene sets were evaluated in terms of completeness by comparing their BUSCO scores with those of three reference mosquito genome assemblies; *A. gambiae*, *A. aegypti*, and *C. quinquefasciatus*.

### Functional Annotation of Gene Sets

Functional annotation of gene sets was performed using eggNOG-mapper v2 (Cantalapiedra et al. 2021) with the following parameters: “-m diamond --sensmode ultra-sensitive --evalue 0.001 --go_evidence all”. Functional annotation of the 11 sand fly gene sets are provided in the Supplementary Material (supplementary file S3, Supplementary Material online).

### Orthology Analysis and Phylogenomics

Orthology analysis between the 11 sand flies and eight other dipteran species was performed using OrthoFinder2 (Emms and Kelly 2019) with the “-M msa” parameter. The genomic resources used in this study are described in the Supplementary Material (supplementary table S5, Supplementary Material online). Species tree estimation was performed as part of the OrthoFinder2 pipeline with FastTree2 on a concatenated alignment of 575 single-copy orthologs. Gene counts of each sand fly species were partitioned according to their orthology profiles using a custom Perl script (classify_orthogroups.pl), and visualized using ggplot2 (Wickham 2016). Orthogroup representation results are also provided in the Supplementary Material (supplementary table S6, Supplementary Material online).

### Phylogenetic Analysis and Visualization

Multiple sequence alignments (MSAs) were performed using MAFFT (Katoh and Standley 2013). MSAs were quality-trimmed using trimAL (Capella-Gutiérrez et al. 2009), and the trimmed alignments were subsequently converted into phylip format. Difficulty of MSAs was assessed using Pythia (Haag et al. 2022). Alignment difficulty was assessed as easy or intermediate for all five studied detoxification gene families; UGTs (0.23), GSTs (0.31), P450s (0.37), ABCs (0.51), and CCEs (0.51). Model selection was performed using ModelTest-NG (Darriba et al. 2020). Maximum likelihood tree reconstruction was performed using RAxML-NG v1.0.2 (Kozlov et al. 2019) with 100 starting trees: 10 random and 90 parsimony trees. The produced phylogenetic tree was midpoint-rooted using the ETE3 toolkit (Huerta-Cepas et al. 2016). Tree visualization was performed using the OliveTRee R package (Charamis 2024), which is based on ggtree (Yu 2020), ape (Paradis and Schliep 2019), and phytools (Revell 2012).

### Manual Curation of Cytochrome P450s in *P. papatasi* and *L. longipalpis* Genomes

Manual curation of cytochrome P450s in the reference *P. papatasi* and *L. longipalpis* genome assemblies (Ppap_2.1; GCA_024763615.2, Llon_2.1; GCF_024334085.1) was performed as described in Dermauw et al. (2020).

### CYP Clusters Across the *P. papatasi* and *L. longipalpis* Genomes

To count CYP genes across the *P. papatasi* and *L. longipalpis* genomes, we implemented a sliding window approach with a window size of 50 kb. CYP genomic clusters were visualized using a custom implementation (gggenomes_lib.R) of the gggenomes R package (Hackl et al. 2023).

### Annotation of Cytochrome P450 Gene Repertoires (CYPomes)

To identify putative sand fly CYP unigenes, we performed DIAMOND (Buchfink et al. 2021) blastp searches (ultra-sensitive mode, *e*-value cutoff: 10^−3^) of the manually curated *P. papatasi* CYPs against each of the sand fly gene sets. The typical P450 protein size is 500 amino acids. We considered CYP unigenes with query coverage of ≥300 amino acids (aa). Then, we manually examined each tree clade and included incomplete CYP unigenes with polypeptide size ≥100 aa, only in the cases of missing orthologs. In the cases of multiple incomplete orthologs, the longest polypeptide sequence was selected. This exhaustive analysis was performed in order to acquire a representative sequence for each CYP ortholog. In the cases in which at least one full-length ortholog was present, all incomplete proteins were considered as either partial transcripts or assembly artifacts and were thus discarded. Therefore, duplications across the gene trees were inferred using only the polypeptides ≥300 aa in size. In the finalized CYPomes, 90.8% (*n* = 1,158) of total genes are ≥300 aa, while 9.2% (*n* = 117) are present as incomplete (100 to 300 aa) (supplementary fig. S4, Supplementary Material online). The 1,275 sand fly CYP polypeptide sequences are provided in the Supplementary Material (supplementary file S6, Supplementary Material online).

### Annotation of Glutathione-S-Transferase (GST) Gene Repertoires

To identify putative sand fly GST unigenes, we performed HMMer searches for the characteristic “PF02798 (Glutathione S-transferase, N-terminal domain)” and “PF00043 (Glutathione S-transferase, C-terminal domain)” domains in each of the sand fly gene sets. *A. gambiae* GST protein size ranges from 200 to 300 aa. We considered GST proteins with query coverage ≥150 aa against the reference database of *A. gambiae* GSTs for inferring gene expansions. We also included five polypeptides with sizes < 150 aa, only in cases of missing orthologs, as we did for CYPs (see above Annotation of Cytochrome P450 Gene Repertoires (CYPomes)). In the final sand fly GST repertoires, 98.20% (*n* = 271) of total unigenes are ≥150 aa, while only 1.80% (*n* = 5) are present as incomplete (100 to 150 aa) (supplementary fig. S5, Supplementary Material online). The 276 sand fly GST polypeptide sequences are provided in the Supplementary Material (supplementary file S8, Supplementary Material online).

### Annotation of UDP-Glucoronosyl/Glucosyl Transferase (UGT) Gene Repertoires

To identify putative sand fly UGT unigenes, we performed HMMer searches for the characteristic “PF00201 (UDP-glucoronosyl and UDP-glucosyl transferase)” in each of the sand fly gene sets. Typically, UGTs have a protein length of 500 aa. Similar to the approach we used for CYPs and GSTs, we considered UGT polypeptides with size ≥300 aa for inferring gene expansions and retained incomplete polypeptides (100 to 300 aa), only when larger orthologs were missing. In the final sets, 83.1% (*n* = 182) of UGT unigenes are ≥300 aa, while 16.9% (*n* = 37) are present as incomplete (100 to 300 aa) (supplementary fig. S6, Supplementary Material online). The 214 sand fly UGT polypeptide sequences are provided in the Supplementary Material (supplementary file S10, Supplementary Material online).

### Annotation of Carboxyl/Cholinesterase (CCE) Gene Repertoires

To identify putative sand fly CCE unigenes, we performed HMMer searches for the characteristic “PF00135 (Carboxylesterase family)” domain in each of the sand fly gene sets. We considered CCE polypeptides with size ≥300 aa and retained incomplete polypeptides (100 to 300 aa), only when larger orthologs were missing. Using this approach, we discarded 140 redundant CCE unigenes, leading to a total 377 CCE unigenes in transcriptome-based sand fly gene sets. Because *Ace1* orthologs were missing from the *P. papatasi* and *P. duboscqi* gene sets, we searched their respective transcriptome assemblies. We identified one incomplete transcript in both species’ transcriptome assemblies, which had *Ace1* as the best hit, and included them in the final dataset. Proteins encoded by both these transcripts clustered with the *Ace1* orthologs in the phylogenetic analysis. In the final sand fly CCE repertoires, 82.1% (*n* = 311) of total unigenes are ≥300 aa, while 17.9% (*n* = 68) are present as incomplete (100 to 300 aa), while the *P. papatasi Ace1* ortholog was smaller than 100 aa in size (supplementary fig. S7, Supplementary Material online). The 379 sand fly CCE polypeptide sequences are provided in the Supplementary Material (supplementary file S12, Supplementary Material online).

### Annotation of ATP-Binding Cassette (ABC) Transporter Gene Repertoires

To identify putative sand fly ABC transporter unigenes, we performed DIAMOND blastp searches (ultra-sensitive mode, *e*-value cutoff: 10^−3^) of the *A. gambiae* set of manually curated ABC transporter genes (Pignatelli et al. 2018) against each of the sand fly gene sets. *A. gambiae* ABC transporter protein sizes range from 435 to >2,000 aa. We considered ABC transporter polypeptides with query coverage ≥ 300 aa, and retained smaller polypeptides, 100 to 300 aa in size, only in cases of missing orthologs. Using this approach, we discarded 93 redundant ABC transporter polypeptides. In the final sets, 93.2% (*n* = 523) are ≥300 aa and 6.7% (*n* = 38) are 100 to 300 aa (supplementary fig. S8, Supplementary Material online). The 561 sand fly CCE polypeptide sequences are provided in the Supplementary Material (supplementary file S14, Supplementary Material online). *Drosophila melanogaster* multidrug resistant (MDR) protein sequences were obtained from FlyBase (Öztürk-Çolak et al. 2024).

### Voltage Gated Sodium Channel (VGSC) Orthologs

We identified conserved orthologs of the *A. gambiae* VGSC (AGAP004707) gene, by parsing the orthology analysis output. MSAs were performed using MAFFT (Katoh and Standley 2013) and visualized using the ggmsa R package (Zhou and Yu 2022). The 11 sand fly VGSC polypeptide sequences and the respective alignment are provided in the Supplementary Material (supplementary files S17 and S18, Supplementary Material online).

### Quantification of Transcript Expression

Transcript expression was quantified using Salmon v.1.9.0 (Patro et al. 2017) in the mapping-based mode. Distribution of gene expression in violin plots was visualized using ggplot2 (Wickham 2016). Normalized gene counts (TPM values) across the 11 sand fly species are provided in the Supplementary Material (supplementary file S19, Supplementary Material online).

## Results

### Assembly and Annotation for 11 Phlebotomine Sand Flies

**Fig. 1.**
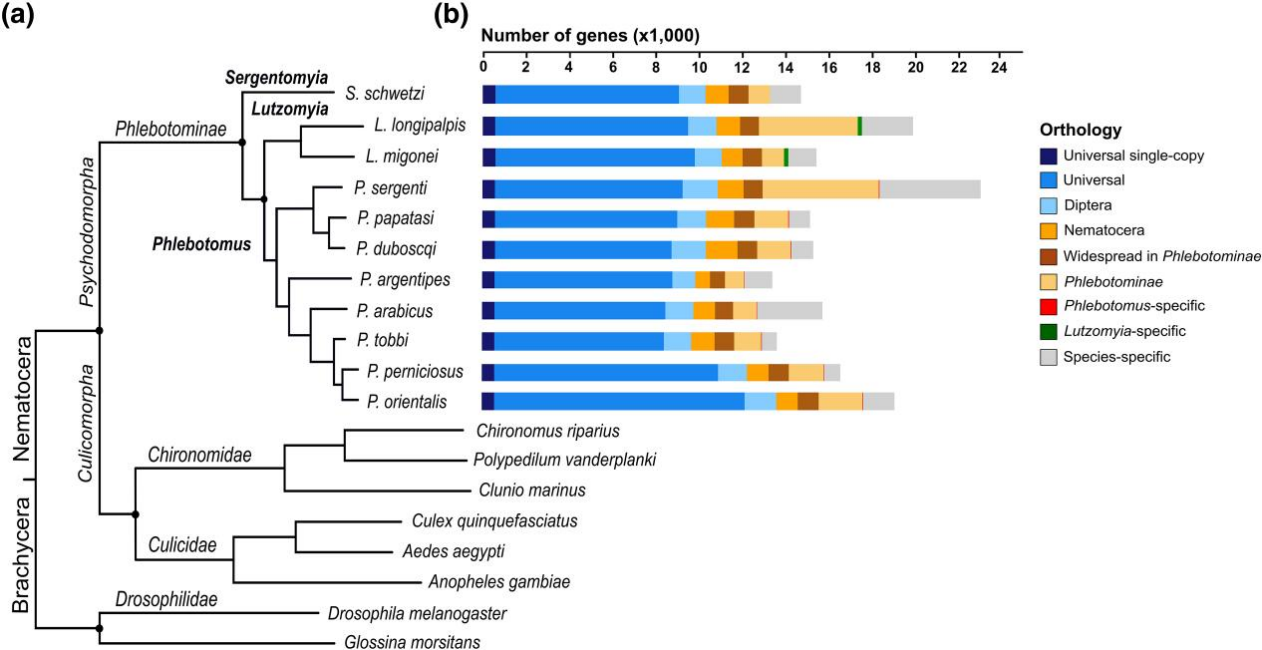
Species phylogeny and gene orthology of the 11 sequenced phlebotomine sand flies. a) Molecular species phylogeny of the 11 sequenced phlebotomines and eight selected dipteran outgroups based on 575 universal single-copy genes. All nodes have 100% bootstrap support. The estimated phylogeny is not time-calibrated. b) Barplots showing gene counts for each species according to their orthology profile, from universal to species-specific genes.

We sequenced and assembled the transcriptomes of 11 phlebotomine sand fly species, eight of which belong to the *Phlebotomus* genus, two traditionally to the *Lutzomyia* genus, and one to the *Sergentomyia* genus (Fig. 1a). In total, we generated more than 1.86 billion Illumina reads, with on average 62 million reads per sample (supplementary table S1, Supplementary Material online).

We performed de novo transcriptome assemblies using Trinity v2.14.0 (Grabherr et al. 2011) and rnaSPADES v3.15.5 (Bushmanova et al. 2019). To evaluate the quality of those assemblies, we employed two distinct quality assessment approaches: a reference-free method with TransRate (Smith-Unna et al. 2016) and a biology-centric method with BUSCO (Manni et al. 2021). These assessments demonstrated that both assemblers produced complete, but also over-assembled transcriptomes (supplementary tables S2 and S3, Supplementary Material online), thus posing potential challenges on the robustness of our comparative genomics approach, which requires both complete and nonredundant sets.

We combined the Trinity and rnaSPADES assemblies (supplementary fig. S1, Supplementary Material online) with Evigene, which uses a multistep approach to detect informative and nonredundant transcripts from over-assembled transcriptomes (Gilbert 2013). BUSCO assessment demonstrated that the combined assemblies exhibit substantially less redundancy and equally high completeness to their parent assemblies (supplementary tables S3 and S4, Supplementary Material online). Transcriptome assembly sequences for each of the 11 phlebotomines are provided in the Supplementary Material (supplementary file S1, Supplementary Material online).

We performed gene prediction using TransDecoder v5.6.0, with integration of homology searches in coding region selection (Haas 2022). Transcriptome assemblies often inflate gene numbers due to biases in alternative splicing, allele differences, and technical sample variation (Guang et al. 2021). To examine the degree to which our gene sets represent distinct loci, we performed CD-HIT clustering (Fu et al. 2012) with varying protein identity cutoffs, and examined gene number changes. We used a 99% cutoff to collapse identical and nearly identical protein isoforms and a 95% cutoff to additionally collapse diverged protein isoforms and recent duplicates. Gene sets produced by combined assemblies (supplementary fig. S2, Supplementary Material online) exhibited relatively small size reductions (medians: 5.7% at CD-HIT 99% and 9.2% at CD-HIT 95%), suggesting that the majority of genes represent independent gene loci. Moreover, these reductions are more than three to five times smaller than those observed for the Trinity (medians: 44.6% at CD-HIT 99% and 55.1% at CD-HIT 95%) and rnaSPADES (medians: 26.2% at CD-HIT 99% and 32.8% at CD-HIT 95%) parent assemblies (supplementary fig. S2, Supplementary Material online), confirming the previously documented redundancy reduction (supplementary tables S3 and S4, Supplementary Material online). To retain a single isoform for each gene, we decided to proceed with gene sets produced by combined assemblies filtered with the 99% protein identity CD-HIT cutoff (supplementary fig. S1, Supplementary Material online).

This transcriptome assembly and gene prediction workflow (supplementary fig. S1, Supplementary Material online) yielded between 12,848 and 22,838 genes (Table 1; median: 14,869), comparable to gene numbers observed in other dipteran species. Differences in gene counts can be primarily attributed to varying numbers of expressed genes in the sequenced samples. Quality of gene annotations was assessed using BUSCO (Manni et al. 2021) on a set of 3,285 conserved dipteran genes (Table 1; supplementary fig. S3, Supplementary Material online). Most of the gene sets produced have high BUSCO completeness (median: 88.4%), with low levels (median: 1.6%) of duplication (Table 1). These scores are relatively comparable to the annotations of three reference mosquito genomes: *Anopheles gambiae*, *Aedes aegypti*, and *Culex quinquefasciatus* (supplementary fig. S3, Supplementary Material online). Protein sequences of predicted gene sets for each of the 11 phlebotomines are provided in the Supplementary Material (supplementary file S2, Supplementary Material online).

**Table 1:** Quality metrics of the 11 sand fly annotations produced in this study

Species	Assembly size (Mb)	Number of genes	Complete BUSCOs (%)	Single-copy BUSCOs (%)	Duplicated BUSCOs (%)	Fragmented BUSCOs (%)	Missing BUSCOs (%)
*P. arabicus*	37.3	15,692	91.1	89.5	1.6	2.4	6.5
*P. argentipes*	29.7	12,848	89.4	87.7	1.7	2.8	7.8
*P. duboscqi*	49.2	14,869	90.9	89.7	1.2	2.3	6.8
*P. orientalis*	42.9	18,788	91.1	81.1	10	2.2	6.7
*P. papatasi*	48.9	14,777	88.9	87.3	1.6	2.9	8.2
*P. perniciosus*	35.6	16,545	86.6	81.3	5.3	4.7	8.7
*P. sergenti*	47.7	22,838	88.7	87.1	1.6	3.3	8
*P. tobbi*	41.3	13,654	90.8	89.6	1.2	1.9	7.3
*L. longipalpis*	44.5	19,619	86	83.8	2.2	4.5	9.5
*L. migonei*	30.5	14,839	79.7	78	1.7	7.6	12.7
*S. schwetzi*	32.8	14,271	89.3	88.2	1.1	2.4	8.3

Last, we functionally annotated 150,364 genes from the 11 sequenced sand fly species using eggNOG-mapper v2 (Cantalapiedra et al. 2021). The pipeline includes searches in the eggNOG 5.0 database of orthology relationships, identification of conserved protein domains, and assignment of GO terms (Cantalapiedra et al. 2021). The eggNOG functional annotations for each of the 11 sand fly gene sets are provided in the Supplementary Material (supplementary file S3, Supplementary Material online).

### Phylogenomics

Orthologous genes are generally considered to have the same function (Gabaldón and Koonin 2013). To examine the patterns of sand fly gene evolution, we performed an orthology analysis of the 11 phlebotomine species with another eight dipteran species (Fig. 1b). This analysis identified 575 universal single-copy orthologs which were used to build the species tree (Fig. 1a). The reconstructed phlebotomine phylogeny is consistent with previously published sand fly species trees (Grace-Lema et al. 2015; Cruaud et al. 2021; Labbé et al. 2023). Most importantly, in agreement with Labbé et al. (2023), this species tree supports clustering of the 11 sand flies with the Nematocera rather than the Brachycera suborder (Fig. 1a).

Among Old World sand fly species included in this study, *P. papatasi* has a vast distribution spanning the Mediterranean basin, including regions of Southern Europe and North Africa, extending across the Middle East and reaching as far as Central Asia and the Indian subcontinent, while *Phlebotomus duboscqi* is distributed across the Sahel region of sub-Saharan Africa. Another vector with vast distribution is *Phlebotomus sergenti*, extending from Canary Islands and Madeira over Portugal, Spain, Maghreb, Sicily, Turkey, Egypt, and Middle East up to Pakistan and India in west–east direction, from southern France to Kenya in north–south direction (Dvořák et al. 2018). *Phlebotomus argentipes* and *Phlebotomus orientalis* are primary vectors of *Leishmania donovani* in the Indian subcontinent and East Africa, respectively. *Phlebotomus arabicus* is distributed in the Middle East and northeastern Africa, and *Phlebotomus perniciosus* and *Phlebotomus tobbi* are found in the Western and Eastern Mediterranean basin, respectively (Dvořák et al. 2018). *Sergentomyia schwetzi* is widely distributed across Africa and transmits leishmaniasis in reptiles but not mammals (Sadlova et al. 2018). The two New World sand fly species included in this study, *L. longipalpis* and *Lutzomyia migonei*, are distributed all across Central and South America (Dvořák et al. 2018). Studies in Argentina have indicated that *L. longipalpis* has a heterogeneous spatial distribution, with limited patches of high abundance (Maroli et al. 2013). Interestingly, *L. migonei* is suspected to be the primary leishmaniasis vector where *L. longipalpis* is absent (Maroli et al. 2013).

Tracing gene content changes relative to an evolutionary lineage can provide insights into the genomic mechanisms underlying lineage-specific adaptations (Thomas et al. 2020). The orthology analysis identified groups of genes with varying representation patterns across the examined 19 dipterans, including universally present genes, as well as genes that are restricted to specific lineages or species (Fig. 1b).

### Evolution of Detoxification Families Across 11 Phlebotomines

To study the evolutionary dynamics of the five major detoxification gene families (CYPs, GSTs, UGTs, CCEs, and ABC transporters) in sand flies, we performed extensive annotation and large-scale phylogenetic comparisons of these families between the 11 sequenced phlebotomines, using *A. gambiae* as reference. This reference species was selected because it is the dipteran vector with the most well-characterized detoxification genes (Ranson et al. 2002). Most CYP, GST, UGT, and CCE groups exhibited complex, many-to-many orthology between sand flies and *A. gambiae*. This is indicative of independent radiations from common ancestral genes. In contrast, one-to-one orthologs were identified between phlebotomines and *A. gambiae* for genes with highly conserved functions such as the ecdysteroidogenic CYPs, non-catalytic CCEs, and the majority of ABC transporters.

#### Annotation of Sand Fly CYP Gene Repertoires (CYPomes)

Cytochrome P450s (CYPs or P450s) constitute the most abundant detoxification enzyme superfamily with a diverse array of functions, ranging from core developmental pathways to xenobiotic metabolism (Dermauw et al. 2020). In total, we annotated 1,275 phlebotomine CYP genes. For *P. papatasi* and *L. longipalpis*, where reference genome assemblies are available (Huang et al. 2024), we manually curated CYP sequences on these assemblies and employed these sequences as representative for downstream analyses. For the remaining nine species, we used CYP sequences derived from transcriptomes. To benchmark the completeness of the transcriptome-based CYPomes, we compared the transcriptome-based with the manually curated CYPomes of *P. papatasi* (*n* = 140) and *L. longipalpis* (*n* = 148). We found that 19 and 38 CYP genes were missing from the transcriptome-based *P. papatasi* (*n* = 121) and *L. longipalpis* (*n* = 110) CYPomes, respectively. Most of the missing genes in *P. papatasi* (17 out of 19) and *L. longipalpis* (27 out of 38) were species-specific and found on a few, gene-rich subfamilies (supplementary table S7, Supplementary Material online). The absence of these genes from the transcriptomes is likely due to lack of, or extremely low, expression in our samples.

#### Most CYP Diversity Is Found in the CYP6, CYP9, and CYP12 Families

**Fig. 2.**
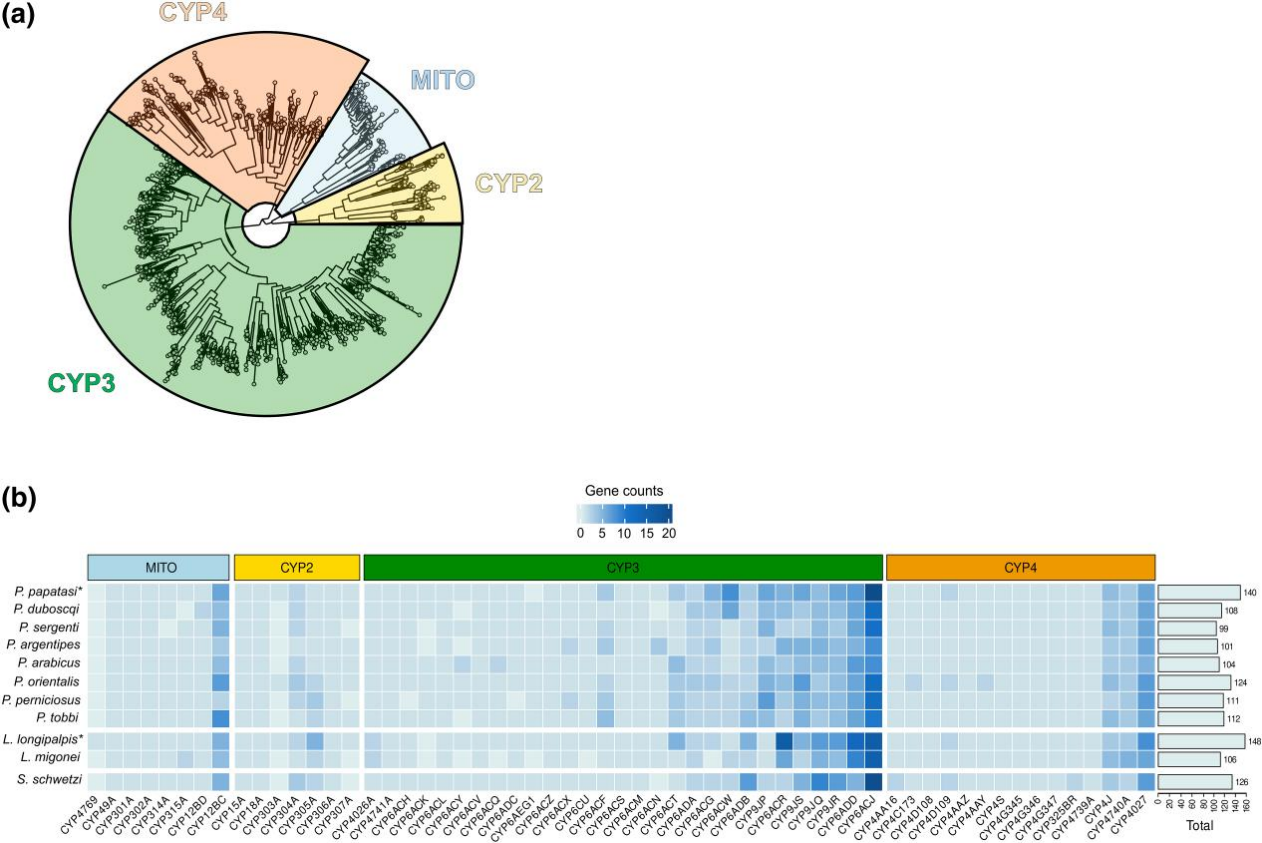
Evolutionary dynamics of cytochrome P450s (CYPs) across 11 phlebotomine sand flies. a) Phylogenetic comparison of 1,275 CYP genes from 11 phlebotomines with *A. gambiae* as reference. Sand fly CYP genes are distributed across the four clans that are typically found in insects. CYP3 clan comprises almost two-thirds of CYP repertoires in sand fly species. b) Gene counts of each sand fly CYP subfamily in the 11 phlebotomine species. *P. papatasi* and *L. longipalpis* gene counts, which are denoted by asterisk (*), were generated by manual curation of CYPs in their reference genome assemblies. Eight CYP6, CYP9, and CYP12 subfamilies are highly expanded and account for most of the CYP variation found between phlebotomines.

Sand fly CYPs are clustered in the four major insect clans; MITO, CYP2, CYP3, and CYP4 clans (Fig. 2a). CYP3 clan comprises almost two-thirds (58% to 64%) of each sand fly CYPome (Fig. 2a), while the CYP6 and CYP9 families accounted for virtually all genes in this clan (Fig. 2b). The 11 sequenced species exhibit high variation in their CYP gene counts, with *P. sergenti* having the fewest (*n* = 99) and *L. longipalpis* the most (*n* = 148) genes (Fig. 2b). Diversity of sand fly CYPomes follows the “Many and the Few” pattern (Dermauw et al. 2020); they consist of many subfamilies with few genes and a few subfamilies with many genes.

**Fig. 3.**
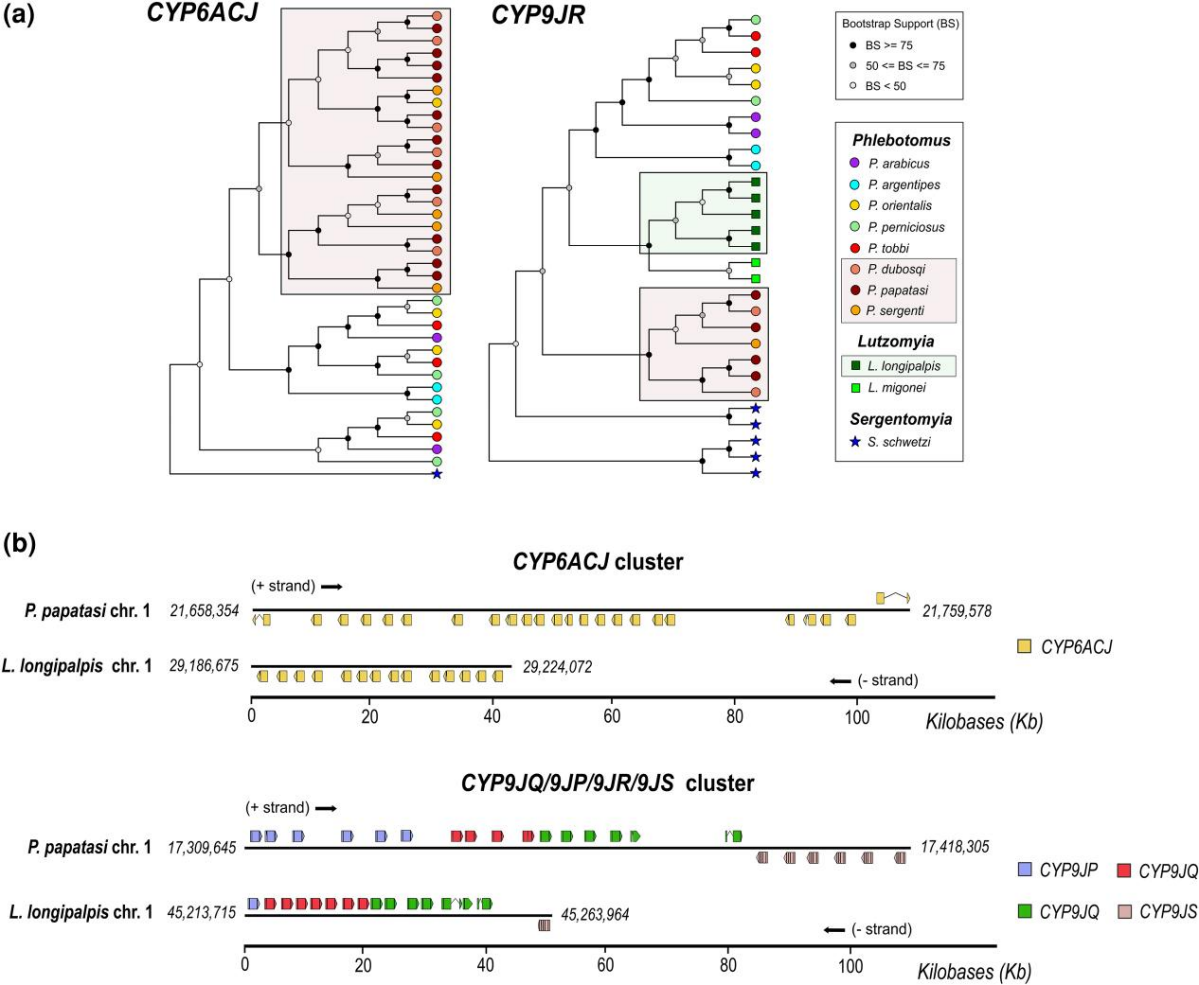
Most CYP variation is attributed to lineage-specific expansions caused by divergent gene amplifications in conserved genomic clusters. a) *CYP6ACJ* and *CYP9JR* orthologs exhibit primarily lineage-specific expansions. Expansions in the *P. papatasi–P. duboscqi–P. sergenti* and *L. longipalpis–L. migonei* lineages are indicated within the boxes. b) CYP6ACJ and CYP9J genes are clustered in the *P. papatasi* and *L. longipalpis* genomes. The clusters have substantially diverged between the two species.

The high end of this unequal distribution is almost exclusively populated by eight gene-rich subfamilies: CYP6ACJ, CYP6ACR, CYP6ADD, CYP9JP, CYP9JQ, CYP9JR, CYP9JS of the CYP3 clan, and CYP12BC of the MITO clan (Fig. 2b). Variation in these subfamilies accounts for most of the total CYP diversity among sand flies (Fig. 2b) and is caused by independent lineage-specific expansions, which mostly occurred after phlebotomine divergence. Here we provide two examples of striking lineage-specific CYP expansions, of CYP6ACJ and CYP9JR genes (Fig. 3a). As illustrated in Fig. 3a, lineage-specific expansions have resulted in 11, 6, and 5 CYP6ACJ genes in *P. papatasi*, *P. duboscqi*, and *P. sergenti*, respectively, while the five *L. longipalpis* CYP9JR genes are species-specific (Fig. 3a).

The emerging pattern of phlebotomine CYP evolution is one of relative stasis for most single-copy genes, followed by concerted lineage-specific expansions for a subset of CYP6, CYP9, and CYP12 orthologs after the split of sand fly lineages (supplementary fig. S9, Supplementary Material online). This raises questions regarding the genomic origins and the potential contribution of these CYP expansions in phlebotomine adaptations to xenobiotics, such as those encountered during the utilization of environmental resources.

#### Divergent Gene Amplifications in Conserved CYP Clusters

CYP families tend to proliferate through multiple lineage-specific gene duplication events, which are known as CYP blooms (Feyereisen 2011) and lead to the formation of clusters in the respective genomes (Dermauw et al. 2020). Therefore, we examined the topology of these eight subfamilies in the two available sand fly genomes, of *P. papatasi* and *L. longipalpis*. We found that almost all members of the CYP6ACJ, CYP6ACR, CYP6ADD, CYP9JP, CYP9JQ, CYP9JR, CYP9JS, and CYP12BC subfamilies are consecutively localized in clusters, but exhibit substantial variation between *P. papatasi* and *L. longipalpis* (Fig. 3b; supplementary fig. S10, Supplementary Material online). As an example, Fig. 3b illustrates the genetic organization of the CYP6ACJ and CYP9JQ/9JP/9JR/9JS clusters in the *P. papatasi* and *L. longipalpis* genomes. A plausible hypothesis is that these CYP clusters originated before the divergence of the *Phlebotomus* and *Lutzomyia* genera, and experienced subsequent expansions following phlebotomine speciation.

P450s involved in xenobiotic metabolism are often part of CYP blooms (Nauen et al. 2022). The eight subfamilies belong to CYP6, CYP9, and CYP12 families, which contain most functionally characterized P450 xenobiotic metabolisers in Diptera (Vontas et al. 2020). The *A. gambiae* (Agam) CYP6, CYP9, and CYP12 families are typically associated with xenobiotic metabolism (Dermauw et al. 2020). More specifically, the sand fly CYP6ACR is most closely related to AgamCYP6AK and exhibits species-specific duplications in *P. argentipes*. CYP6ADD is most closely related to AgamCYP6AG1 and is most highly expanded in the two *Lutzomyia* species (Fig. 2b). CYP6ACJ is most closely related to the AgamCYP6P subfamily (supplementary fig. S9, Supplementary Material online). Lineage-specific duplications of CYP6ACJ orthologs are common (Fig. 3a) and have led to striking gene gains in *P. papatasi*, *L. longipalpis*, and *S. schwetzi* (Fig. 2b). CYP9JP, CYP9JQ, and CYP9JR are similar to AgamCYP9J/9L. Notably, CYP9JP exhibits species-specific expansions in *P. papatasi*, while CYP9JQ and CYP9JR exhibit species-specific expansions in *L. longipalpis* and *S. schwetzi*. CYP9JS is orthologous to AgamCYP9K1, a functionally validated deltamethrin metabolizer (Vontas et al. 2020). Finally, CYP12BC is orthologous to AgamCYP12F. Taken together, the genomic and phylogenetic evidence suggest that phlebotomines exhibit multiple independent lineage-specific expansions in CYPs related to known xenobiotic metabolisers.

We also identified CYP6ACW and CYP6ACX, which are phylogenetically related to the AgamCYP6M and AgamCYP6P subfamilies, respectively. These two subfamilies include two well-characterized anopheline pyrethroid metabolizers, AgamCYP6M2 and AgamCYP6P3 (Vontas et al. 2020). Notably, CYP6ACW displays lineage-specific gene gains in *P. papatasi* (*n* = 8) and *P. duboscqi* (*n* = 6).

#### Phlebotomines Have Two Conserved CYP4G17 Orthologs

CYP4 is the second largest clan, making up about one-quarter of sand fly CYPomes (Fig. 2b). Phlebotomines have 23 to 31 genes in this clan, considerably less than *A. gambiae* which has 43. This is mostly due to fewer CYP4AAY, CYP4C, and CYP325 genes.

**Fig. 4.**
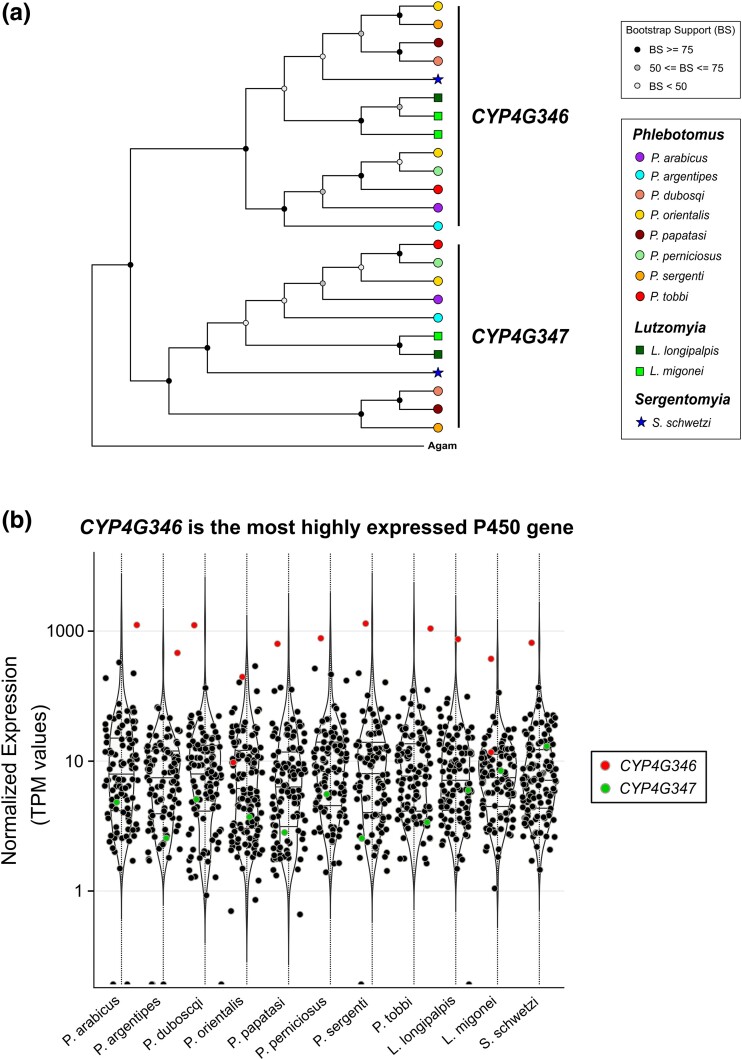
Sand flies have two conserved orthologs of CYP4G17, an important enzyme involved in cuticular hydrocarbon biosynthesis. a) Phylogenetic comparison of *CYP4G17* orthologs in 11 phlebotomine species with *A. gambiae* (Agam) as reference. b) Violin plots of mean log10-transformed normalized expression values (transcripts per million) of total CYP genes on each of the 11 sequenced phlebotomines *CYP4G346* orthologs are consistently the most highly expressed CYP genes in each species, while *CYP4G347* orthologs are much less expressed. This indicates that among the two, *CYP4G346* is the functional ortholog of *CYP4G17* in sand flies.

There are two main CYP4G clades in insects, *CYP4G1* and *CYP4G15*, named after the genes initially characterized in *Drosophila melanogaster* (Feyereisen 2020). *CYP4G17* is the single CYP4G1 ortholog in *A. gambiae* and encodes for an oxidative decarbonylase catalyzing an essential step in cuticular hydrocarbon biosynthesis (Kefi et al. 2019). Suggestive of its crucial physiological role, *CYP4G1* is also the most highly expressed CYP gene in *D. melanogaster* (Feyereisen 2020). Here we identified two phlebotomine-conserved orthologs of *CYP4G17*, named *CYP4G346* and *CYP4G347* (Fig. 4a). *CYP4G346* is consistently the most highly expressed CYP gene across phlebotomines, more than 100 times compared to *CYP4G347* (Fig. 4b). This indicates that among the two, *CYP4G346* is the functional ortholog of *CYP4G17*. Duplications of CYP4G1 genes are not unusual and have occurred independently in several dipteran lineages (Feyereisen 2020). Despite their well-characterized role in insect hydrocarbon biosynthesis, a direct link between CYP4G gene copy number and associated phenotypes, such as increased cuticular hydrocarbon content or enhanced desiccation tolerance, has not been established yet (Feyereisen 2020). CYP4G16 orthologs are single-copy in phlebotomines, as is the case for all Diptera sequenced to date (Feyereisen 2020).

#### Orthologs for Highly Conserved CYPs of the MITO and CYP2 Clans

With the exception of CYP12, MITO and CYP2 families have a relatively stable number of genes between sand flies and *A. gambiae* (Fig. 2b). We also found one-to-one orthologs for highly conserved CYPs that perform key functions in regulating the biosynthesis and activity of core insect hormones. These include P450s involved in the hydroxylation (CYP302A1, CYP306A1, CYP315A1) of ecdysteroid precursors and activation (CYP314A1) of ecdysone to 20-hydroxyecdysone (20E). CYP307, a component of 20E biosynthesis, is found in seven phlebotomines, but was not found in the transcriptomes of five species (supplementary table S7, Supplementary Material online). Similarly, orthologs for CYP303A1, which was recently demonstrated to be essential for embryonic development and adult eclosion in *D. melanogaster* (Wu et al. 2019), were absent from 8 out of 11 transcriptomes. The absence of these genes likely results from their very low expression in adults. Significantly, both genes are found in the genome assemblies of *P. papatasi* and *L. longipalpis* (supplementary table S7, Supplementary Material online). Unlike *A. gambiae*, phlebotomines have a CYP18A1 ortholog, a conserved P450 enzyme that catalyzes ecdysone inactivation (Guittard et al. 2011). Furthermore, all 11 sand flies have a CYP15 ortholog, the enzyme that carries out juvenile hormone (JH) epoxidation in mosquitoes (Nouzova et al. 2021).

#### *Lutzomyia*-Specific Expansions of GSTD and GSTX

**Fig. 5.**
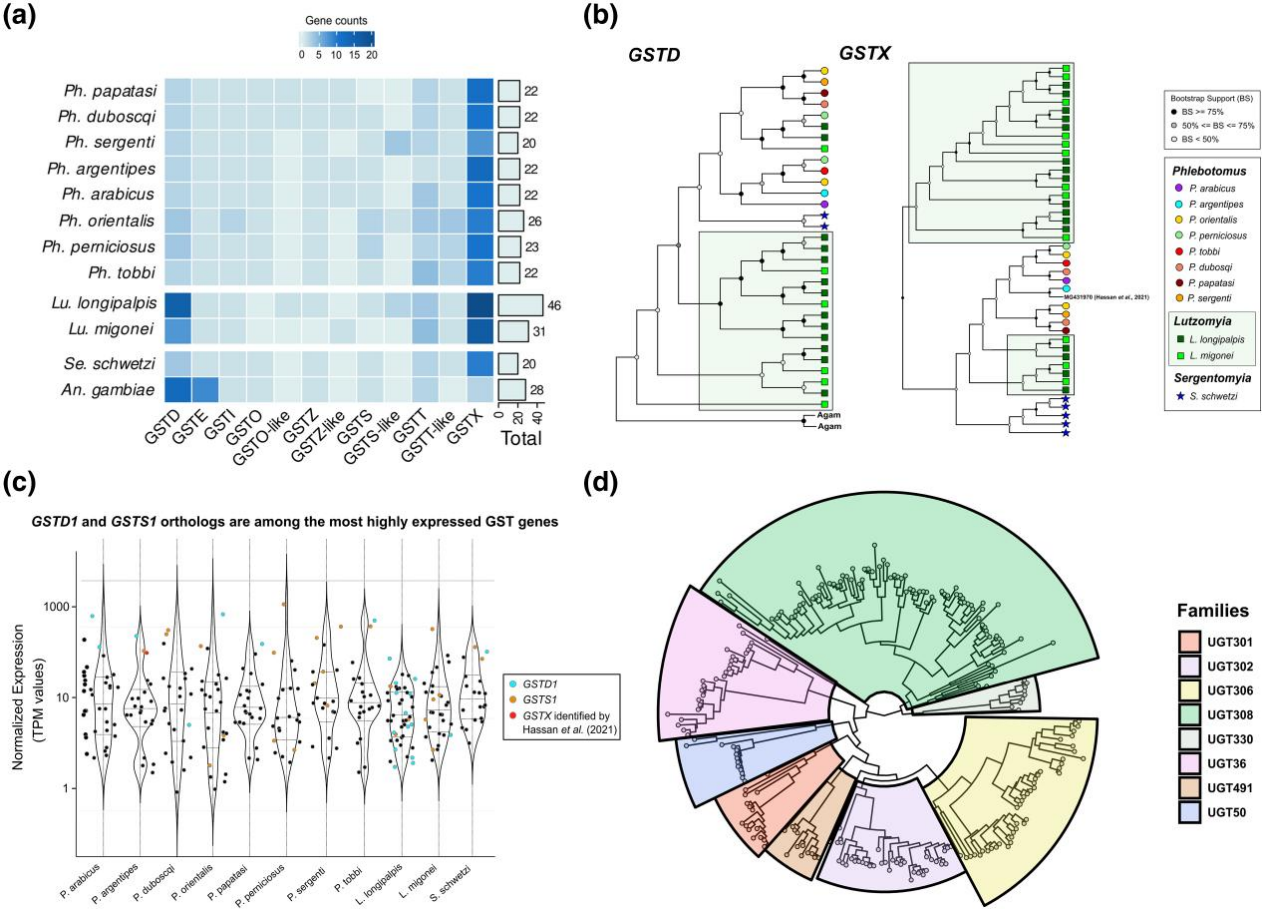
New World sand flies exhibit striking lineage-specific expansions of GSTX and GSTD genes. a) Gene counts for each GST class across the 11 phlebotomines. GSTX is the most abundant class in all phlebotomines, while GSTD has expanded in the two *Lutzomyia* species. b) GSTD and GSTX orthologs display *Lutzomyia*-specific expansions, while minor changes are observed in the other phlebotomine species. *Lutzomyia*-specific expansions are indicated within the boxes. The functionally characterized *P. argentipes* GST, previously labeled as GSTD by Hassan et al. (2021), is actually a GSTX ortholog. c) Violin plots of mean log10-transformed normalized expression values (transcripts per million) of total GST genes on each of the 11 sequenced phlebotomines. *GSTD1* and *GSTS1* orthologs are consistently among the most highly expressed GST genes. In *P. argentipes*, the functionally characterized GSTX (Hassan et al. 2021) is the third most highly expressed GST gene. d) Phylogenetic distribution of 276 phlebotomine UGTs into eight families with *A. gambiae* as reference.

Arthropod GSTs comprise a large enzyme family involved in the detoxification of chemical compounds such as plant allelochemicals, insecticides, and byproducts of oxidative stress (Koirala et al. 2022). Our analysis identified 276 sand fly GST genes, most of which belong to the eight classes found in mosquitoes and other dipterans (Lumjuan et al. 2007). We also discovered three conserved sand fly clades, which have no orthologs with the *A. gambiae* GST genes and are basal to the GSTO, GSTZ, GSTS, and GSTT classes, respectively (supplementary fig. S11, Supplementary Material online). We refer to those classes as GSTO-like, GSTZ-like, GSTS-like, and GSTT-like, respectively (Fig. 5a; supplementary table S8, Supplementary Material online).

The number of GST genes is similar between *Phlebotomus* species (*n* = 20 to 26) and *S. schwetzi* (*n* = 20), while *L. longipalpis* (*n* = 46) and *L. migonei* (*n* = 31) exhibit notable expansions (Fig. 5a). GSTX is the most abundant class in all 11 sand flies (Fig. 5a). Phlebotomines have 7 to 20 GSTX genes, many more than anophelines which have only two (Neafsey et al. 2015). Variation between phlebotomines is also found due to lineage-specific expansions of GSTX paralogs, particularly in *L. longipalpis* and *L. migonei* (Fig. 5b). Phylogenetically, GSTX is related to the insect-specific GSTD and GSTE classes, which are typically involved in xenobiotic detoxification and insecticide resistance in mosquitoes (Pavlidi et al. 2018). Moreover, a GSTX gene from *P. argentipes* was recently shown to metabolize DDT in vitro (Hassan et al. 2021). This gene (GenBank accession: MG431970.3) was originally characterized as a member of the GSTD class by Hassan et al. (2021). However, a phylogenetic analysis of this sequence with our extensive sand fly GST dataset demonstrated that this is in fact a GSTX, instead of a GSTD gene (Fig. 5b; supplementary fig. S11, Supplementary Material online). Interestingly, it is the third most highly expressed GST gene in *P. argentipes* (Fig. 5c).

Phlebotomine GSTD genes are paralogs of *GSTD7* and *GSTD1*. Interestingly, we identified substantial *Lutzomyia*-specific expansions of the *GSTD1* paralogs (Fig. 5b). GSTD genes, particularly orthologs of *GSTD1*, have been linked to insecticide metabolism and resistance in mosquitoes and other arthropods (Pavlidi et al. 2018). Therefore, these data suggest that *L. longipalpis* and *L. migonei* display expansions of GST genes related to xenobiotic metabolizers.

Sand flies have a single conserved *GSTE* gene, which is an ortholog of the anopheline *GSTE8*. GSTE8 was recently involved in ecdysteroidogenesis in *A. gambiae* (Musdal et al. 2023). A GSTS gene (GenBank accession: MG431969.1) was recently implicated in *P. argentipes* DDT resistance. The study provided evidence suggesting that this GSTS protein mediates inducible increased stress tolerance after DDT exposure (Hassan et al. 2019). By comparing this gene with our extended GST dataset, we identified this gene as the conserved *P. argentipes* ortholog of *A. gambiae GSTS1* (supplementary fig. S11, Supplementary Material online). Interestingly, *GSTS1* and *GSTD1* orthologs ranked consistently as the most highly expressed GST genes across all 11 phlebotomines (Fig. 5c). Moreover, the DDT-metabolizing GSTX identified by Hassan et al. (2021) ranked as the third most abundant GST gene in *P. argentipes* (Fig. 5c). We also identified conserved orthologs for *GSTI1* which was recently implicated in insect adaptation to xenobiotics (Koirala et al. 2022).

#### Independent Radiations of UGT Families in Phlebotomines

UGTs are phase II conjugating enzymes with a crucial role in xenobiotic detoxification, including insecticide metabolism, as well as in the modulation of signaling pathways (Ahn and Marygold 2021). Our analysis identified 214 sand fly UGT genes which cluster to eight families (Fig. 5d; supplementary table S9, Supplementary Material online). Phlebotomines have 15 to 25 UGT genes, less than that of *A. gambiae* (*n* = 26). UGT308, UGT306, UGT36, and UGT302 are the most abundant families (Fig. 5d) and exhibit many-to-many orthology between phlebotomines and *A. gambiae* (supplementary fig. S12, Supplementary Material online). Diversity between sand flies is constrained. Most notably, UGT308 displays an expansion in *L. longipalpis* (supplementary fig. S12, Supplementary Material online). We also quantified expression of UGT genes across the 11 phlebotomines (supplementary fig. S13, Supplementary Material online). Despite being extensively studied in mammals, our understanding of the physiological role and molecular function of UGTs in arthropods remains limited (Ahn and Marygold 2021). Genes belonging to the UGT308 and UGT302 families have been previously found overexpressed in insecticide resistant mosquitoes (Zhou et al. 2019).

#### A Relative Paucity of Catalytic α-esterases

**Fig. 6.**
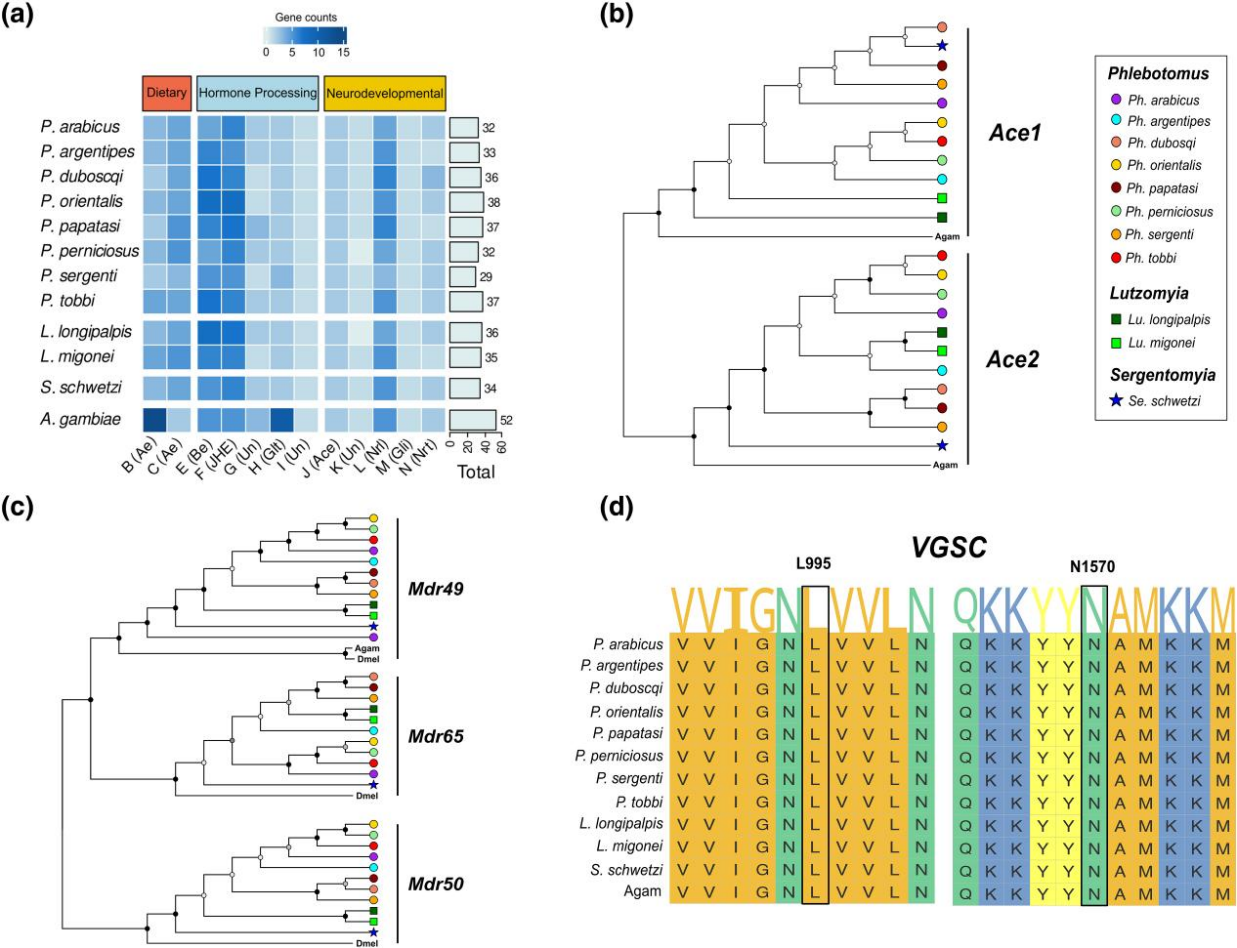
Conserved one-to-one orthologs for genes involved in pesticide toxicity. a) Gene counts of each CCE clade across the 11 phlebotomines. Sand flies have substantially less glutactin and α-esterase genes. b) Phlebotomines have conserved one-to-one orthologs for both acetylcholinesterase *A. gambiae* (Agam) genes, *Ace1* and *Ace2*. Ace1 is the target of organophosphate and carbamate insecticides in *A. gambiae*. c) Sand flies have conserved orthologs for all three *D. melanogaster* (*Dmel*) ABCB Full Transporter (FT) genes, including *Mdr65* and *Mdr50* which are missing from *A. gambiae* (Agam). Bootstrap support and species symbols used are the same as in (b). ABCB FTs play a crucial role in pesticide transport and toxicity as previously demonstrated in the fruit fly *D. melanogaster*. d) Multiple sequence alignment of the voltage gated sodium channel (VGSC) gene across 11 phlebotomine sand fly species and *A. gambiae* (Agam). VGSC is a known target of pyrethroid insecticides and the regions shown harbor the two most important resistance-conferring mutations. These regions were identified as pyrethroid-sensitive across all 11 sequenced phlebotomines.

Carboxyl/cholinesterases (CCEs) are involved in host plant adaptation, pesticide resistance as well as insect olfaction (Cruse et al. 2023). We identified 379 sand fly CCEs which cluster into 13 major clades and three main functional classes based on the classification provided by Oakeshott et al. (2010) (Fig. 6a; supplementary fig. S14, Supplementary Material online). Phlebotomines have substantially less CCE genes (*n* = 32 to 38) than those found in *A. gambiae* (*n* = 52), while diversity among sand flies is limited (Fig. 6a; supplementary table S10, Supplementary Material online). Dipteran α-esterases are divided in two clades, B and C (Oakeshott et al. 2010). We found only half as many α-esterases in phlebotomines compared to *A. gambiae*, due to substantially fewer clade B genes (Fig. 6a). In contrast, clade C α-esterases and β-esterases (clade E) are equally abundant in phlebotomines (Fig. 6a). We also observed substantially less glutactin (Glt) genes in all sand flies compared to *A. gambiae* (Fig. 6a).

#### Conserved Orthologs for *Ace1* and *Ace2*

Importantly, we identified conserved one-to-one orthologs for both mosquito acetylcholinesterase genes, *Ace1* and *Ace2* (Fig. 6b). *Ace1* is a crucial neurotransmitter regulator and the target of organophosphate and carbamate insecticides in mosquitoes (Grau-Bové et al. 2021). We also identified one-to-one orthologs for neuroligins, glioactins, and neurotactins (supplementary fig. S14, Supplementary Material online), membrane-bound proteins that lack catalytic activities and are functionally associated with Ace (Cruse et al. 2023). Examination of CCE gene expression across the 11 phlebotomines did not provide additional insights into the function of these genes (supplementary fig. S15, Supplementary Material online).

#### Conserved Orthologs for ABC Transporters Involved in Pesticide Toxicity

ABC transporters are ubiquitous transmembrane proteins that transport a large diversity of endogenous and exogenous substrates across lipid membranes, with multiple functions in arthropods including xenobiotic transport (Dermauw and Van Leeuwen 2014). Sand fly species have a similar number (*n* = 45 to 55) and distribution (subfamilies A to H) of ABC genes to that of *A. gambiae* (*n* = 53). Most ABC genes are identified as one-to-one orthologs between each phlebotomine and *A. gambiae*, while expansions are limited (supplementary table S11, Supplementary Material online; supplementary fig. S16, Supplementary Material online). We also examined the expression of ABC transporter genes across the 11 phlebotomines. Our analysis revealed that orthologs of *ABCE1*, *ABCF1*, and *ABCG15* consistently ranked among the most highly expressed ABC transporter genes (supplementary fig. S17, Supplementary Material online). ABCE and ABCF transporters play a role in translation regulation (Amezian et al. 2024). The high expression levels of the *ABCE1* ortholog observed here align with a potentially essential role, consistent with findings in *Tenebrio molitor* (Dermauw and Van Leeuwen 2014). Additionally, ABCFs have been shown to be among the most highly expressed ABC transporter genes in *A. gambiae* females (Pignatelli et al. 2018). ABCG transporters are well-known for their involvement in the transport of lipids and steroids (Dermauw and Van Leeuwen 2014).

Our findings also demonstrate the presence of conserved phlebotomine orthologs for the Mdr49, Mdr50, and Mdr65 ABCB full transporters which are known to be involved in absorption and excretion of xenobiotics (Denecke et al. 2022). Unlike mosquitoes and nonbiting midges (Liu et al. 2021), phlebotomines have conserved orthologs for *Mdr50* and *Mdr65* (Fig. 6c). Genetic studies in *D. melanogaster* have established Mdr65 as a predominant ABC transporter involved in chemoprotection against pesticides, while the role of Mdr49 and Mdr50 in xenobiotic transport has also been documented (Denecke et al. 2022). Furthermore, all phlebotomines have conserved orthologs for ABCH2 (supplementary fig. S16, Supplementary Material online), which was recently shown to act as a key modulator of deltamethrin toxicity in *Anopheles coluzzii* (Kefi et al. 2023).

#### Voltage Gated Sodium Channel (VGSC) Orthologs

The voltage gated sodium channel (VGSC), a crucial enzymatic component of the insect nervous system, is the primary target of pyrethroid insecticides and DDT (Clarkson et al. 2021). In this study, we identified conserved one-to-one orthologs of the *A. gambiae VGSC* gene in sand flies and investigated the presence of mutations at two conserved positions known to confer target-site pyrethroid resistance (Clarkson et al. 2021): L995 and N1570 (the codon numbering given here is relative to transcript AGAP004707-RD, as defined in the AgamP4.14 gene-set annotations) (Figure 6d), also known as L1014F and N1575Y, respectively (Dong et al. 2014). Both of these sites were detected as pyrethroid-sensitive in the 11 sand fly species (Fig. 6d). The lack of resistance-conferring mutations from our dataset is not unexpected, as the sequenced samples were derived from susceptible laboratory-reared populations.

## Discussion

Research related to sand fly adaptations to xenobiotics has been hampered by the limited availability of genomic resources. Here we sequenced and assembled the transcriptomes of 11 sand fly species, thus greatly expanding the available genomic resources for those important disease vectors.

Decisions in transcriptome assembly strategy strongly affect the quality of downstream comparative analyses and the biological conclusions deduced from them (Guang et al. 2021). To enable high quality comparative genomics analyses, our transcriptome assembly and gene prediction strategy aimed at producing complete and nonredundant gene sets. We decided to use two assemblers, as recent studies have demonstrated that no single assembler performs best in all situations (Hölzer and Marz 2019; Voshall et al. 2021). We selected Trinity and rnaSPADES which were previously recognized to consistently produce good assemblies across diverse biological datasets (Hölzer and Marz 2019). In our study, both assemblers produced complete but also redundant transcriptomes, as demonstrated by the TransRate and BUSCO assessments (supplementary tables S2 and S3, Supplementary Material online). Combining the output of different assemblers has been recently suggested to produce more comprehensive assemblies, as some degree of complementarity seems to exist among genes missed by different assembly algorithms (Hölzer and Marz 2019). Rather than selecting the output of a single assembler, we decided to combine the Trinity and rnaSPADES outputs using Evigene (supplementary fig. S1, Supplementary Material online). This approach significantly reduced redundancy in the combined assemblies while preserving the high completeness of the parent assemblies (supplementary table S4, Supplementary Material online).

While our analyses indicate that most of our predicted genes represent single loci (supplementary fig. S2, Supplementary Material online), some degree of over- or under-estimation is inevitable in transcriptome assemblies, due to their inherent biases in expression, transcript coverage, and splice variant annotation (Freedman et al. 2021). To mitigate the effects of such biases on our downstream analyses, we carefully annotated more than 2,700 detoxification genes from the five main detoxification families (CYPs, GSTs, UGTs, CCEs, and ABC transporters) of the 11 sequenced phlebotomines. Especially for CYPs which is the most abundant, variable and difficult-to-assemble detoxification gene family, we also manually curated the gene repertoires on the available *P. papatasi* and *L. longipalpis* genome assemblies, and used them to benchmark our transcriptome-based CYPomes. Subsequently, we thoroughly examined the evolutionary dynamics of the five detoxification families across 11 phlebotomines using *A. gambiae* as reference. These patterns reflect how detoxification has evolved in sand flies and is suggestive of candidate genes potentially involved in their adaptations to xenobiotics.

Notwithstanding limitations of transcriptome-based datasets, the main findings from each comparative gene family analysis are robustly supported by non-fragmented representatives from multiple sand fly species, while for CYPs they are also corroborated by genome-based analyses. We found that most variation among the 11 phlebotomines is found in eight CYP subfamilies and two GST classes. These eight CYP subfamilies (CYP6ACJ, CYP6ACR, CYP6ADD, CYP9JP, CYP9JQ, CYP9JR, CYP9JS, and CYP12BC) are related to known and potential CYP metabolizers, and have expanded in multiple lineages after phlebotomine divergence. Similarly, GSTD and GSTX are phylogenetically related to functionally validated GST metabolizers and display striking lineage-specific expansions in *L.* (*Lutzomyia*) *longipalpis* and *L.* (*Migonemyia*) *migonei*.

Whether these CYP and GST expansions are primarily adaptive or neutral remains an open question (Dermauw et al. 2020), as gene family expansions often occur and are maintained as a result of nonadaptive evolutionary forces (Feyereisen 2011). An array of in vivo and in vitro experimental studies, such as RNAi or CRISPR-Cas9 knockouts (Douris et al. 2020; Nauen et al. 2021), toxicity bioassays, and biochemical assays (Shi et al. 2023) should be performed to examine the potential role of these CYPs and GSTs in sand fly adaptations to plant-derived and chemically-produced xenobiotics. Nonetheless, this comparative study identified groups of CYP and GST genes with promising candidates for future molecular studies on xenobiotic adaptations, such as insecticide resistance, in sand flies.

Furthermore, we identified sand fly one-to-one orthologs of *Ace1* and *VGSC*, the primary targets of organophosphate/carbamate (Grau-Bové et al. 2021) and DDT/pyrethroid (Clarkson et al. 2021) insecticides, respectively. Previous studies have also identified a conserved single-copy *Ace1* ortholog in *P. papatasi* (Temeyer et al. 2014) and *L. longipalpis* (Coutinho-Abreu et al. 2007), and biochemically characterized its potential for developing well-known target-site resistance mutations. In addition, our analysis revealed the absence of resistance-conferring mutations in all 11 sand fly VGSC orthologs, a finding consistent with the insecticide susceptible phenotype of these laboratory strains. Recent monitoring studies have suggested that *kdr* (L995F and L995S) mutations are typically absent from *Phlebotomus* species in Greece and Italy (Balaska et al. 2023). Nonetheless, they have been recorded in *P. argentipes*, *P. papatasi*, and *P. tobbi* field-collected samples from relevant eco-epidemiological settings in Armenia (Paronyan et al. 2024), Turkey, India, and Sri Lanka (Balaska et al. 2021; Reid et al. 2023). Annotating the sand fly orthologs of this critical insecticide target will contribute to the development of innovative molecular diagnostic assays (Paronyan et al. 2024), facilitating the efficient monitoring of insecticide resistance mutations in sand flies.

Overall, the evolutionary insights and genomic resources produced in this study provide a foundation for elucidating the molecular mechanisms underlying sand fly adaptations to either naturally-occurring or chemically-produced xenobiotics.

## Supplementary Material

evae186_Supplementary_Data

## Data Availability

Raw sequencing reads are deposited in SRA under BioProject PRJNA1055492, while the 11 sand fly transcriptome assemblies are deposited in NCBI TSA under the following accessions: *P. arabicus* (SUB14323108), *P. argentipes* (SUB14323321), *P. duboscqi* (SUB14323319), *P. orientalis* (SUB14323352), *P. papatasi* (SUB14323354), *P. perniciosus* (SUB14323363), *P. sergenti* (SUB14323366), *P. tobbi* (SUB14323373), *L. longipalpis* (SUB14323341), *L. migonei* (SUB14323350), and *S. schwetzi* (SUB14323380). The 11 sand fly transcriptome assemblies and gene sets are also available at FigShare (https://doi.org/10.6084/m9.figshare.25459042.v5) together with the other 17 Supplementary Files. The code used in this study is also available at FigShare (https://doi.org/10.6084/m9.FigShare.25480774.v3) and GitHub (https://github.com/JasonCharamis/SandFlyComparativeGenomics).
