# Supplementary material for: Comparative Genomics Uncovers the Evolutionary Dynamics of Detoxification and Insecticide Target Genes Across 11 Phlebotomine Sand Flies
Source: Genome Biol Evol. 2024 Sep 3;16(9):evae186. doi: 10.1093/gbe/evae186 (PMC11412322; doi:10.1093/gbe/evae186)
Supplement: evae186_Supplementary_Data [file evae186_supplementary_data.zip › PROOFS_GBE-240417.R1_Supplementary_Figures.pdf]

Supplementary Material for:

Comparative genomics uncovers the evolutionary dynamics of detoxification and insecticide target genes across 11 phlebotomine sand flies

Jason Charamis[1,2,*], Sofia Balaska[1,2], Panagiotis Ioannidis[2,3], Vít Dvořák[4], Konstantinos Mavridis[2], Mary Ann McDowell[5], Pavlos Pavlidis[1,3], René Feyereisen[6], Petr Volf[4], John Vontas[2,7,*]

[1]Department of Biology, University of Crete, Vassilika Vouton, 71409, Heraklion, Greece.

[2]Institute of Molecular Biology and Biotechnology, Foundation for Research and Technology-Hellas, 70013, Heraklion, Greece.

[3]Institute of Computer Science, Foundation for Research and Technology-Hellas, Heraklion, Greece.

[4]Department of Parasitology, Faculty of Science, Charles University, Prague, Czech Republic.

[5]Eck Institute for Global Health, Department of Biological Sciences, University of Notre Dame, Notre Dame, IN, USA.

[6]Laboratory of Agrozoology, Department of Plants and Crops, Faculty of Bioscience Engineering, Ghent University, Coupure links 653, 9000, Ghent, Belgium.

[7]Pesticide Science Laboratory, Department of Crop Science, Agricultural University of Athens, 11855, Athens, Greece.

*Corresponding authors: E-mails: jason_charamis@imbb.forth.gr; vontas@imbb.forth.gr.

Table of Contents

Supplementary Figures S1-17 ... 3

Supplementary Figure S1. ... 3

Supplementary Figure S2. ... 4

Supplementary Figure S3. ... 5

Supplementary Figure S4. ... 6

Supplementary Figure S5. ... 7

Supplementary Figure S6. ... 8

Supplementary Figure S7. ... 9

Supplementary Figure S8. ... 10

Supplementary Figure S9. ... 11

Supplementary Figure S10. ... 12

Supplementary Figure S11. ... 13

Supplementary Figure S12. ... 14

Supplementary Figure S13. ... 15

Supplementary Figure S14. ... 16

Supplementary Figure S15. ... 17

Supplementary Figure S16. ... 18

Supplementary Figure S17. ... 19

Supplementary Table Legends S1-11 ... 20

Supplementary Figures S1-17

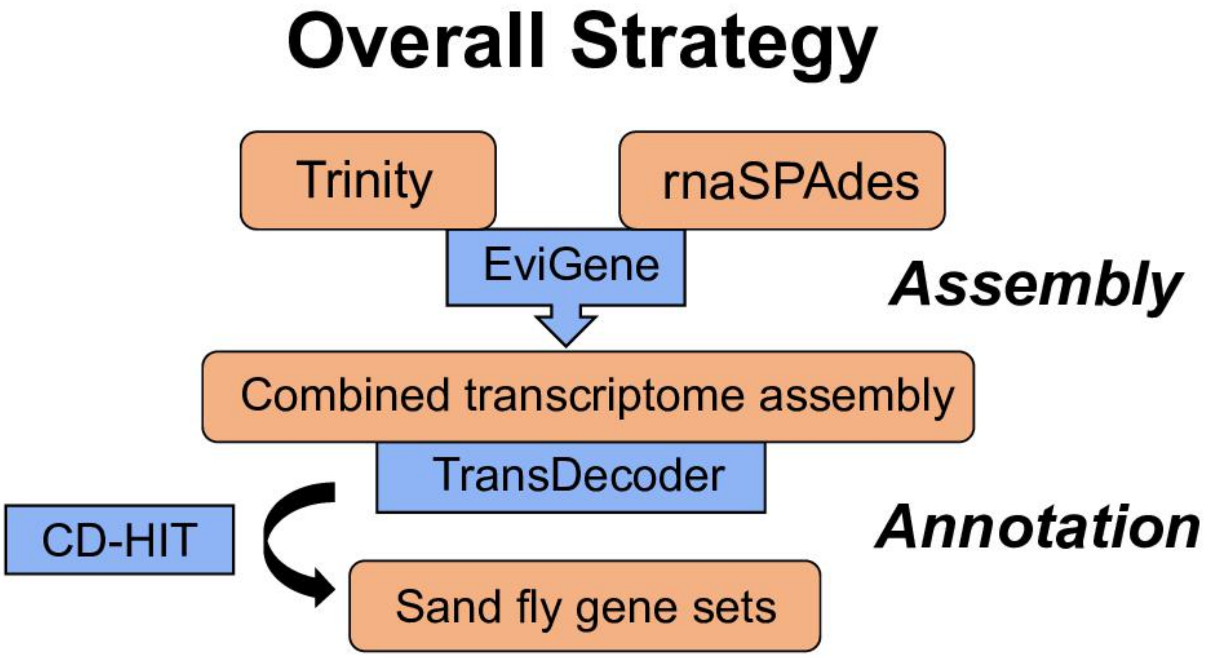

Supplementary Figure S1. Transcriptome assembly and annotation workflow used in this study.

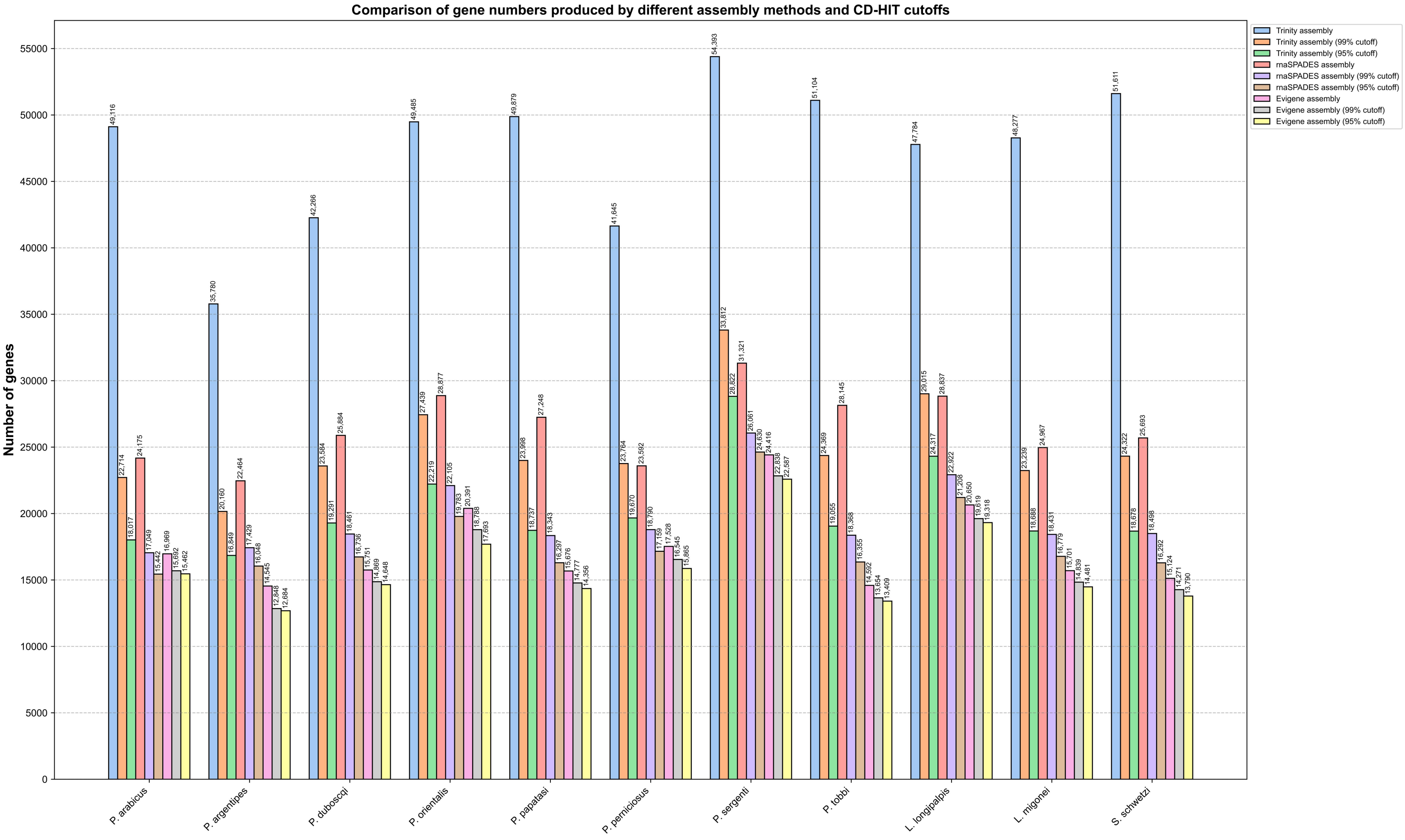

Supplementary Figure S2. Gene number changes in gene sets produced by Trinity, rnaSPADES and Evigene transcriptome assemblies without CD-HIT filtering, and with 99% and 95% protein identity cutoffs.

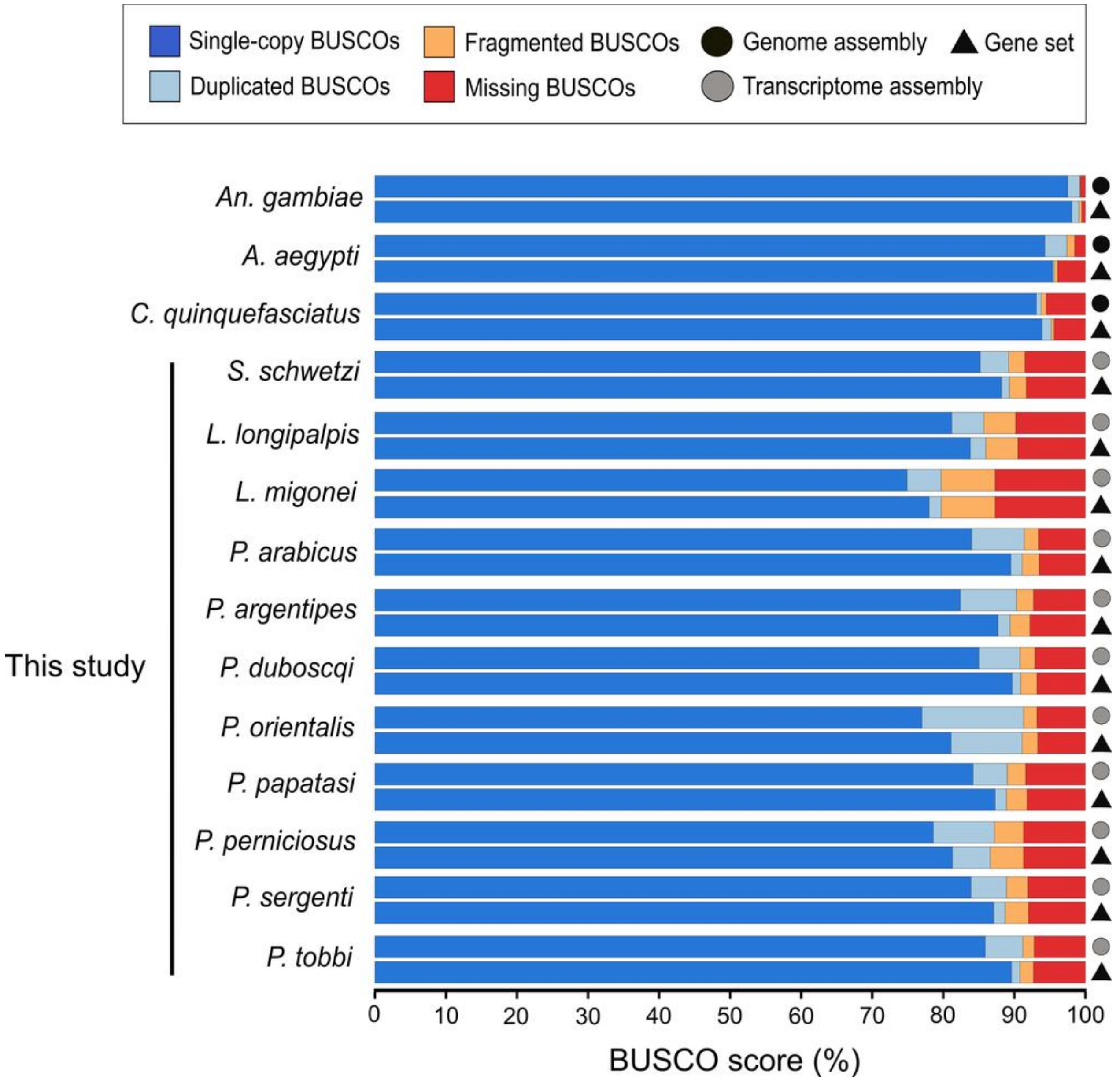

Supplementary Figure S3. BUSCO scores of the 11 sand fly transcriptome assemblies and gene sets produced in this and those of three reference mosquito species: *Anopheles gambiae*, *Aedes aegypti* and *Culex quinqefasciatus*.

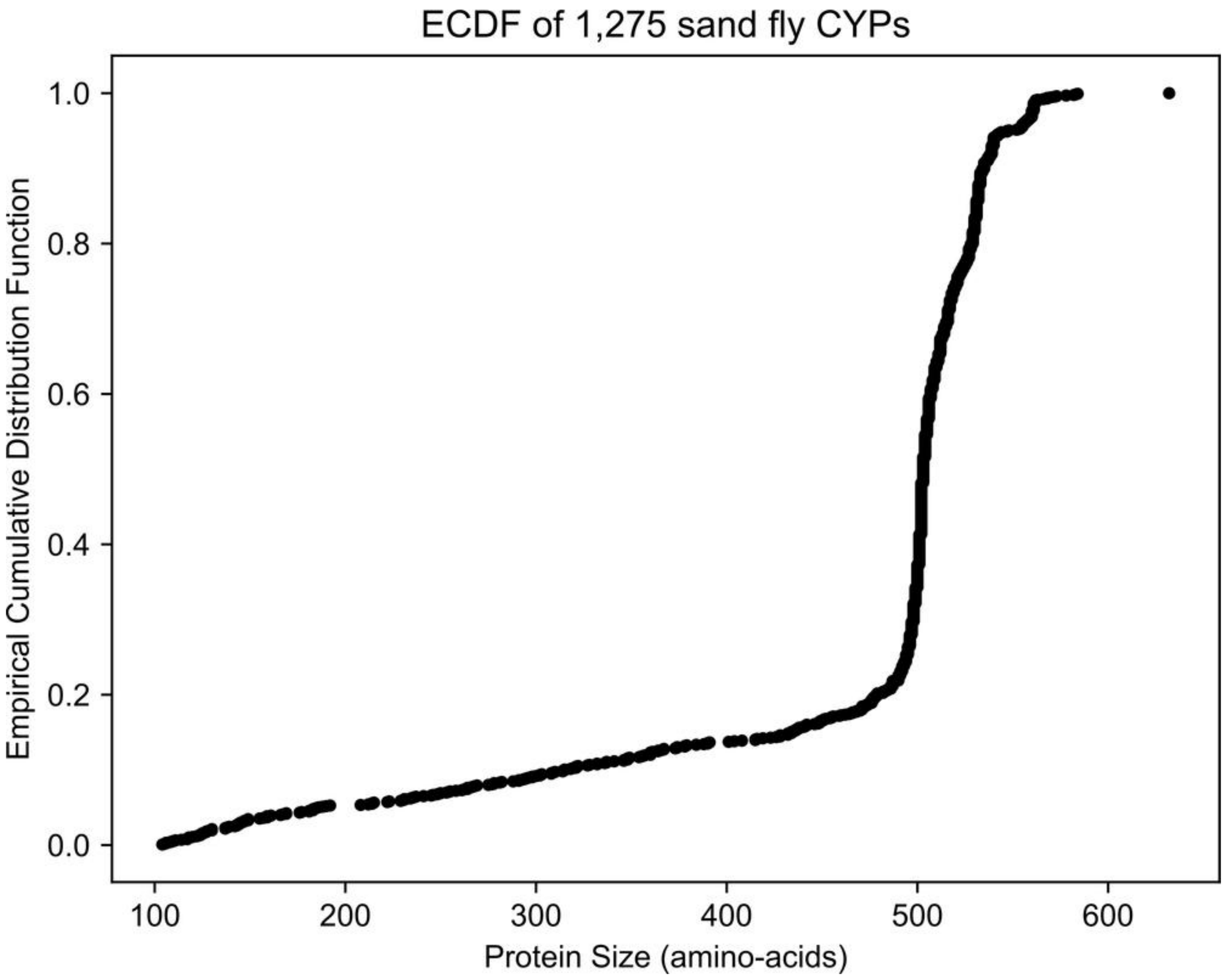

Supplementary Figure S4. Empirical Cumulative Distribution (ECDF) of 1,275 sand fly CYPs. More than 96% of the total CYP polypeptides are >= 150 amino-acids.

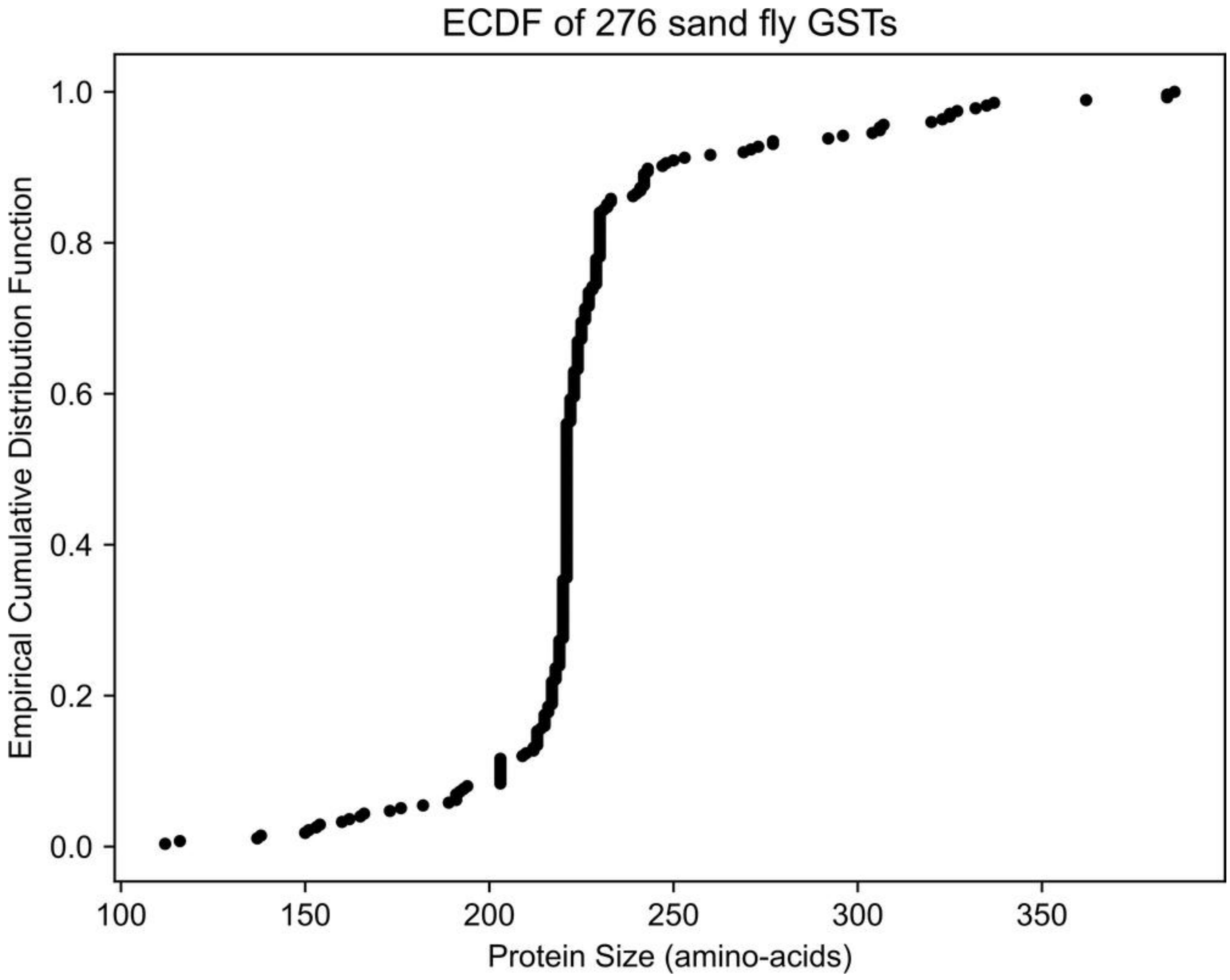

Supplementary Figure S5. Empirical Cumulative Distribution (ECDF) of 276 sand fly GSTs. More than 98% of the total GST polypeptides are >= 150 amino-acids.

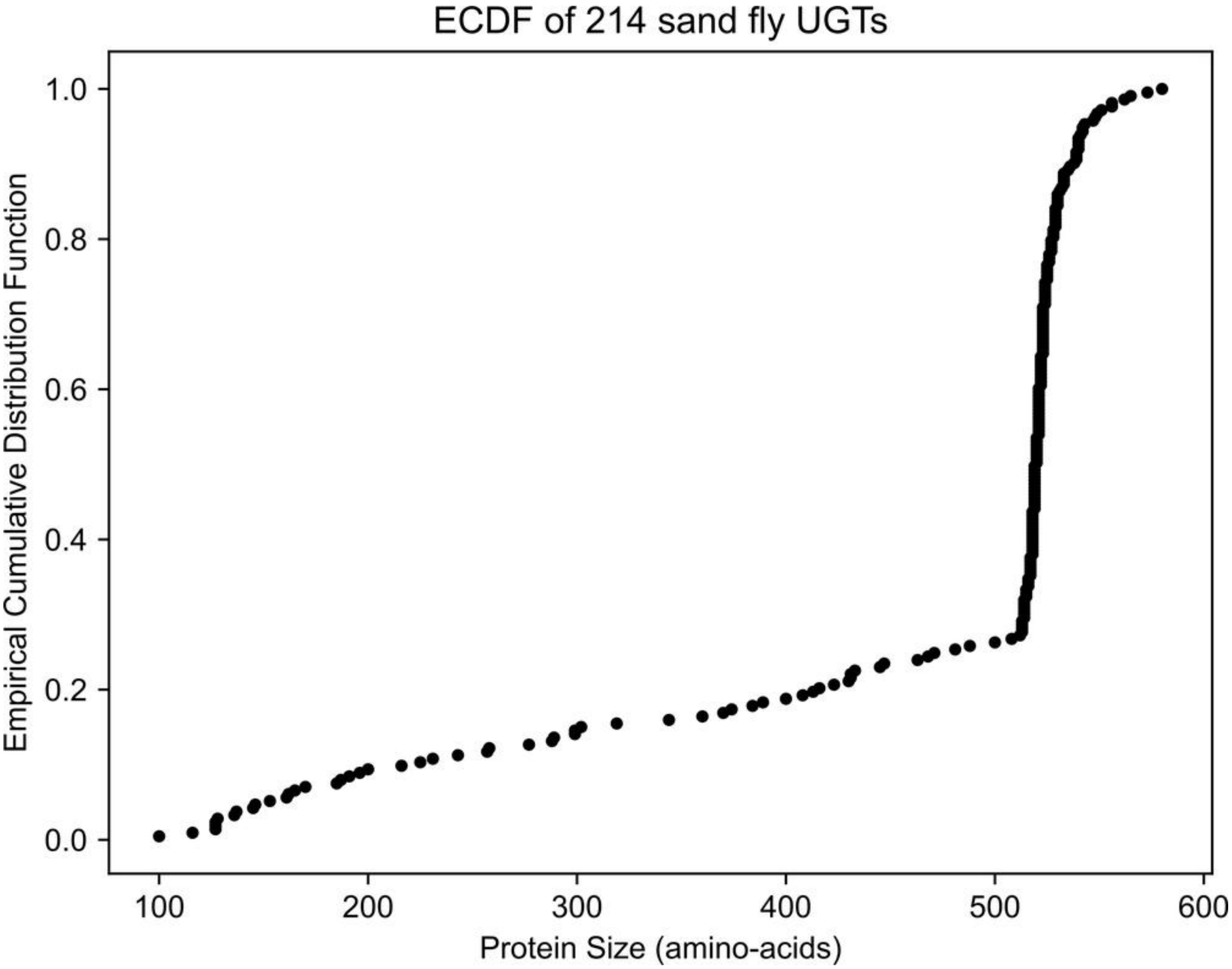

Supplementary Figure S6. Empirical Cumulative Distribution (ECDF) of 214 sand fly UGTs. More than 94% of the total UGT polypeptides are >= 150 amino-acids.

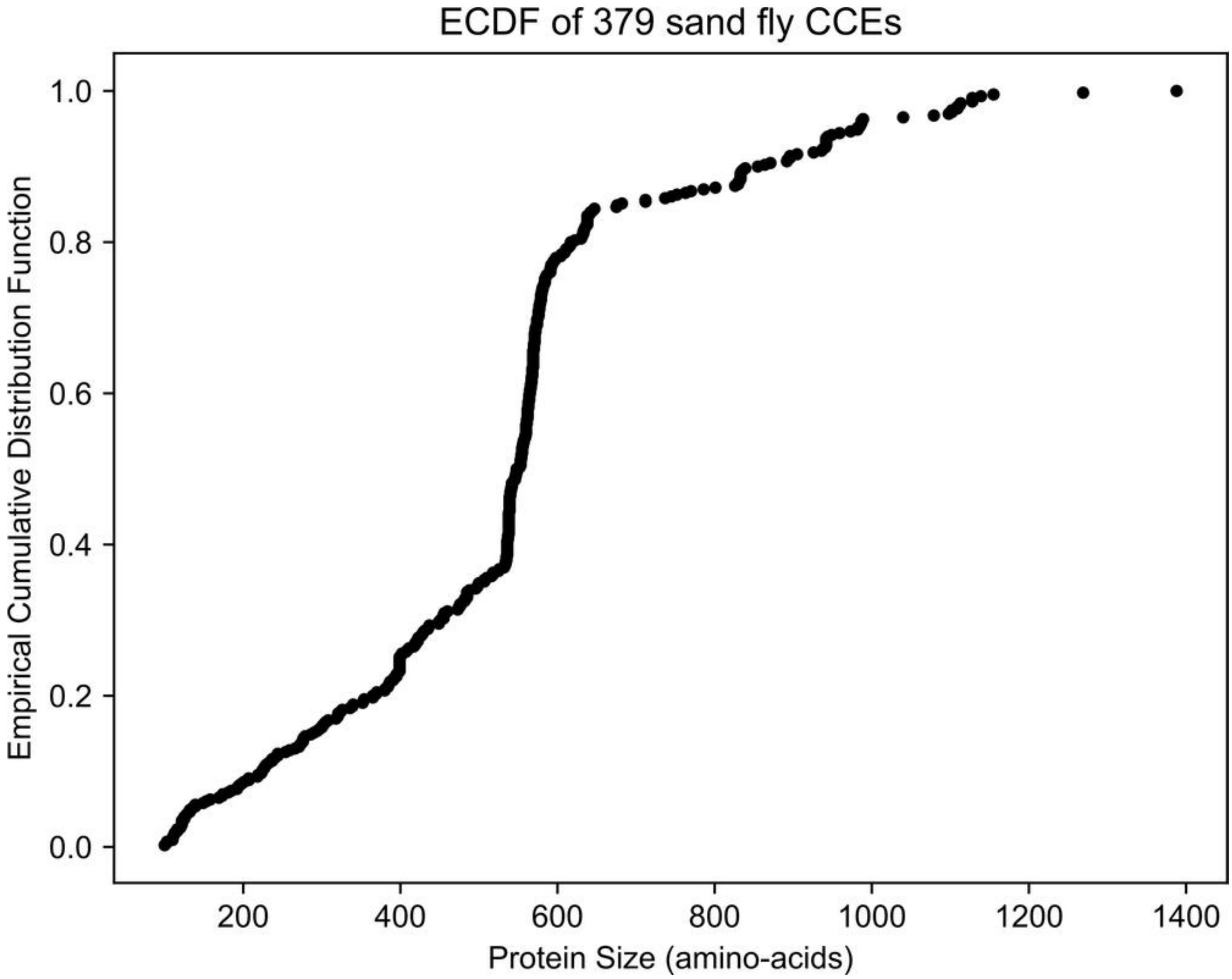

Supplementary Figure S7. Empirical Cumulative Distribution (ECDF) of 379 sand fly CCEs. More than 93% of the total CCE polypeptides are >= 150 amino-acids.

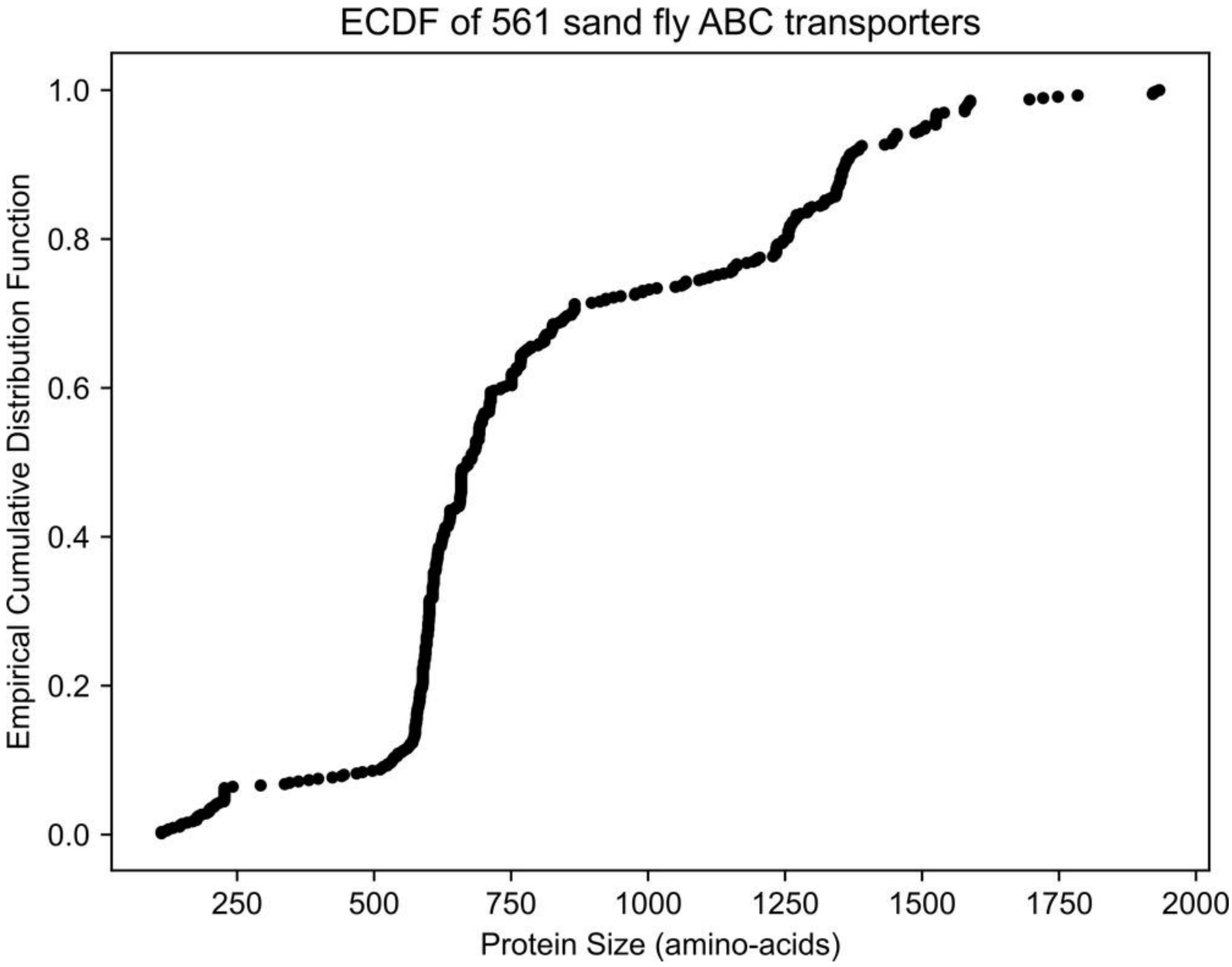

Supplementary Figure S8. Empirical Cumulative Distribution (ECDF) of 561 sand fly ABC transporters. More than 98.5% of the total ABC transporter polypeptides are >= 150 amino-acids.

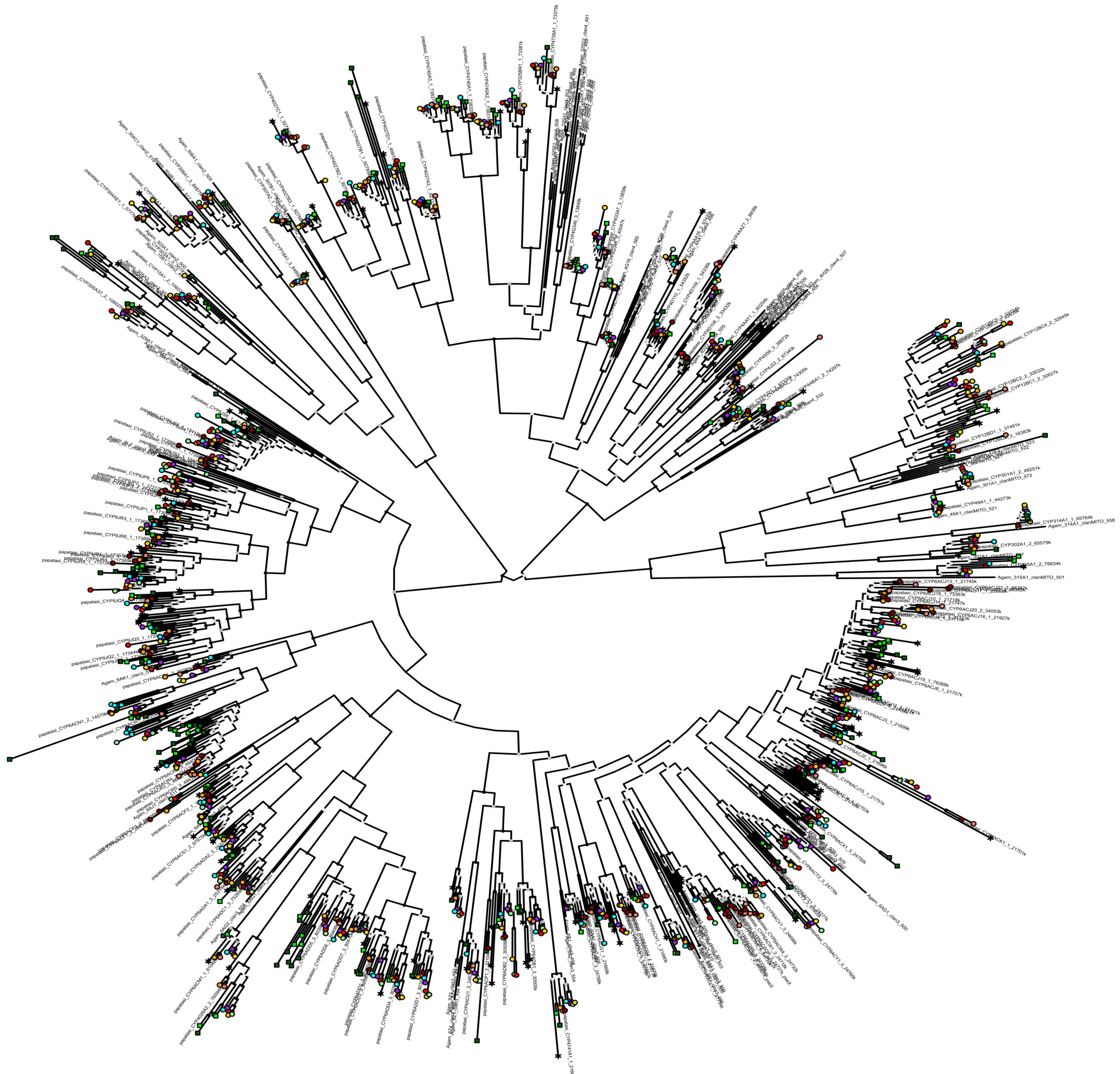

Supplementary Figure S9. Maximum likelihood phylogeny of 1,275 sand fly P450s using *A. gambiae* (n=100) as reference. Branches are colored based on their corresponding P450 clan: cyan (MITO clan), gold (CYP2 clan), green (CYP3 clan) and orange (CYP4 clan). Sand fly species are denoted using a combination of color and shapes: *P. arabicus* (purple circle), *P. argentipes* (light cyan circle), *P. duboscqi* (brown circle), *P. orientalis* (gold circle), *P. perniciosus* (turquiose circle), *P. sergenti* (orange circle), *P. tobbi* (red circle), *L. longipalpis* (dark green square), *L. migonei* (light green square) and *S. schwetzi* (blue star). The *P. papatasi* CYP genes are provided using their official CYP names. Bootstrap values are denoted with light grey (<50% bootstrap support), grey (50-75% bootstrap support) and black (>75% bootstrap support) circles.

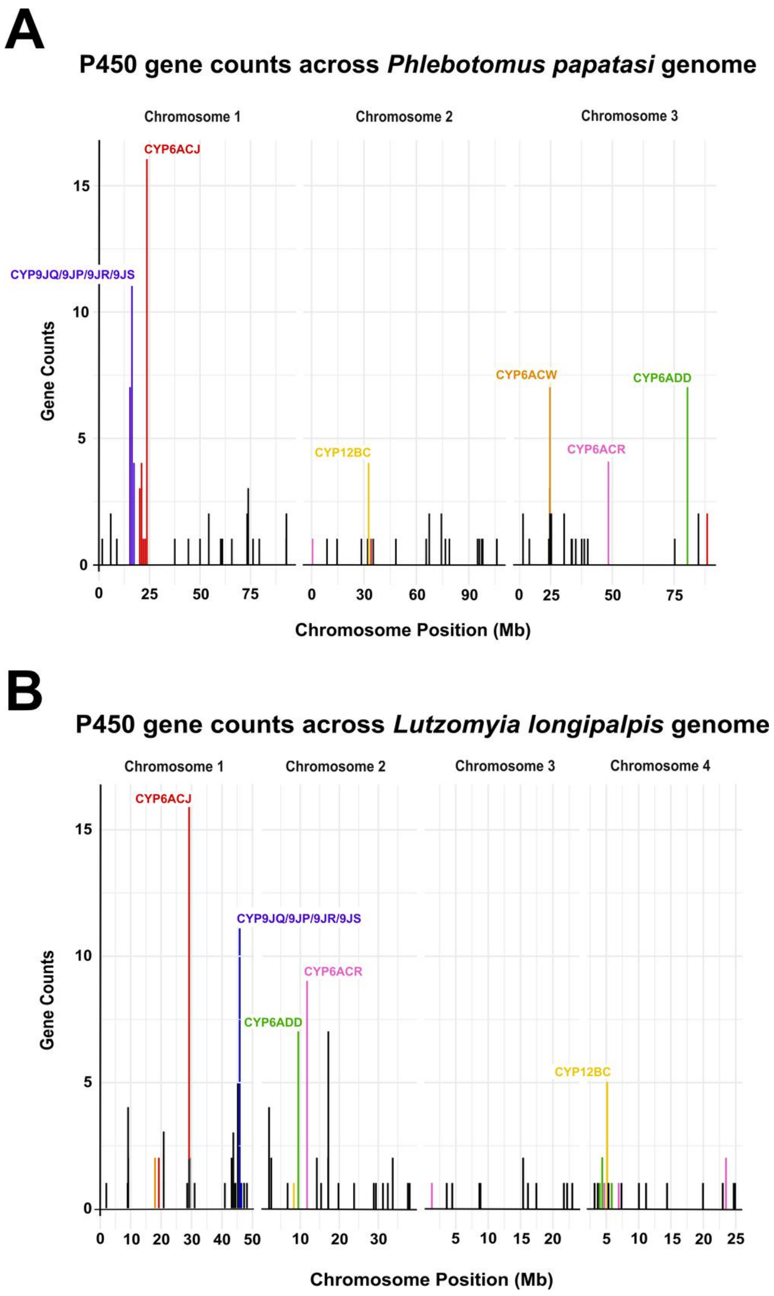

Supplementary Figure S10. CYP gene counts across the *P. papatasi* (A) and *L. longipalpis* (B) genomes using a sliding window of 50-kb size. Most of the CYP6ACJ, CYP6ACR, CYP6ADD, CYP9JP, CYP9JQ, CYP9JR, CYP9JS and CYP12BC genes (colored) are found in genomic clusters.

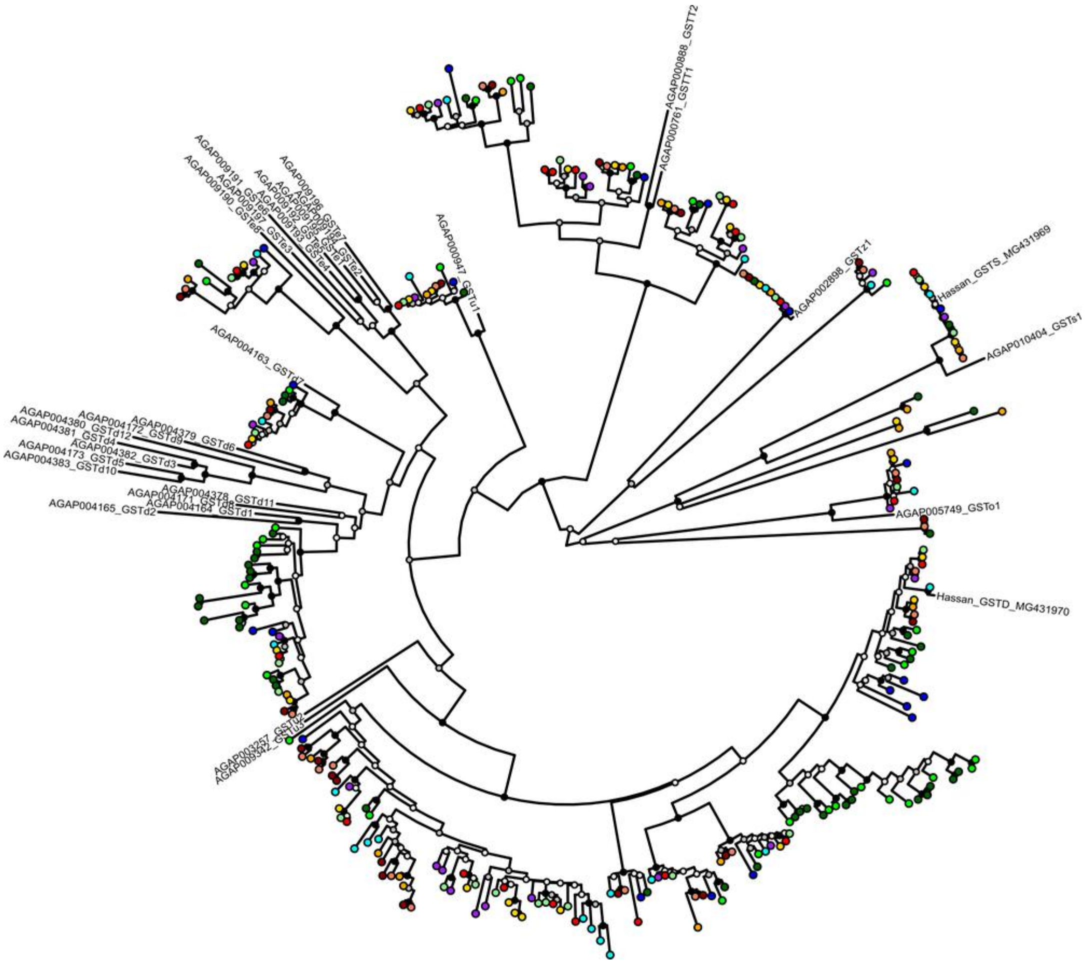

Supplementary Figure S11. Maximum likelihood phylogeny of 276 sand fly GST genes and the two *P. argentipes* GSTs functionally characterized by Hassan et al. (2019) and Hassan et al. (2021), with *A. gambiae* (n=28) as reference. Sand fly species are denoted using a combination of color and shapes: *P. arabicus* (purple circle), *P. argentipes* (light cyan circle), *P. duboscqi* (brown circle), *P. orientalis* (gold circle), *P. papatasi* (dark red circle), *P. perniciosus* (turquiose circle), *P. sergenti* (orange circle), *P. tobbi* (red circle), *L. longipalpis* (green square), *L. migonei* (light green square) and *S. schwetzi* (blue star). Bootstrap values are denoted with light grey (<50% bootstrap support), grey (50-75% bootstrap support) and black (>75% bootstrap support) circles.

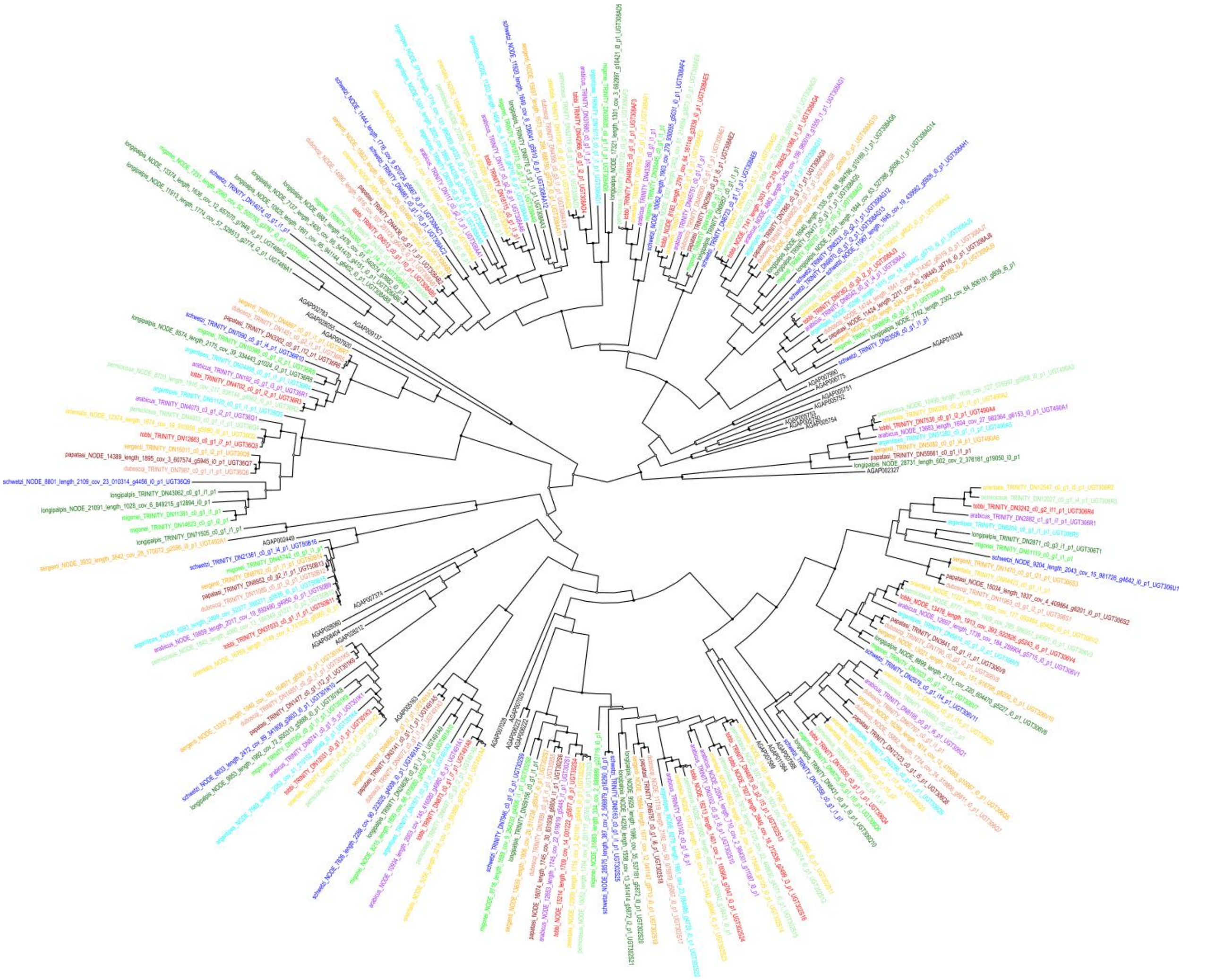

Supplementary Figure S12. Maximum likelihood phylogeny of 214 sand fly UGT genes with *A. gambiae* (n=26) as reference. Species are also denoted using color: *P. arabicus* (purple), *P. argentipes* (light cyan), *P. duboscqi* (brown), *P. orientalis* (gold), *P. papatasi* (dark red), *P. perniciosus* (turquiose), *P. sergenti* (orange), *P. tobbi* (red), *L. longipalpis* (dark green), *L. migonei* (light green) and *S. schwetzi* (blue). Bootstrap values are denoted with light grey (<50% bootstrap support), grey (50-75% bootstrap support) and black (>75% bootstrap support) circles.

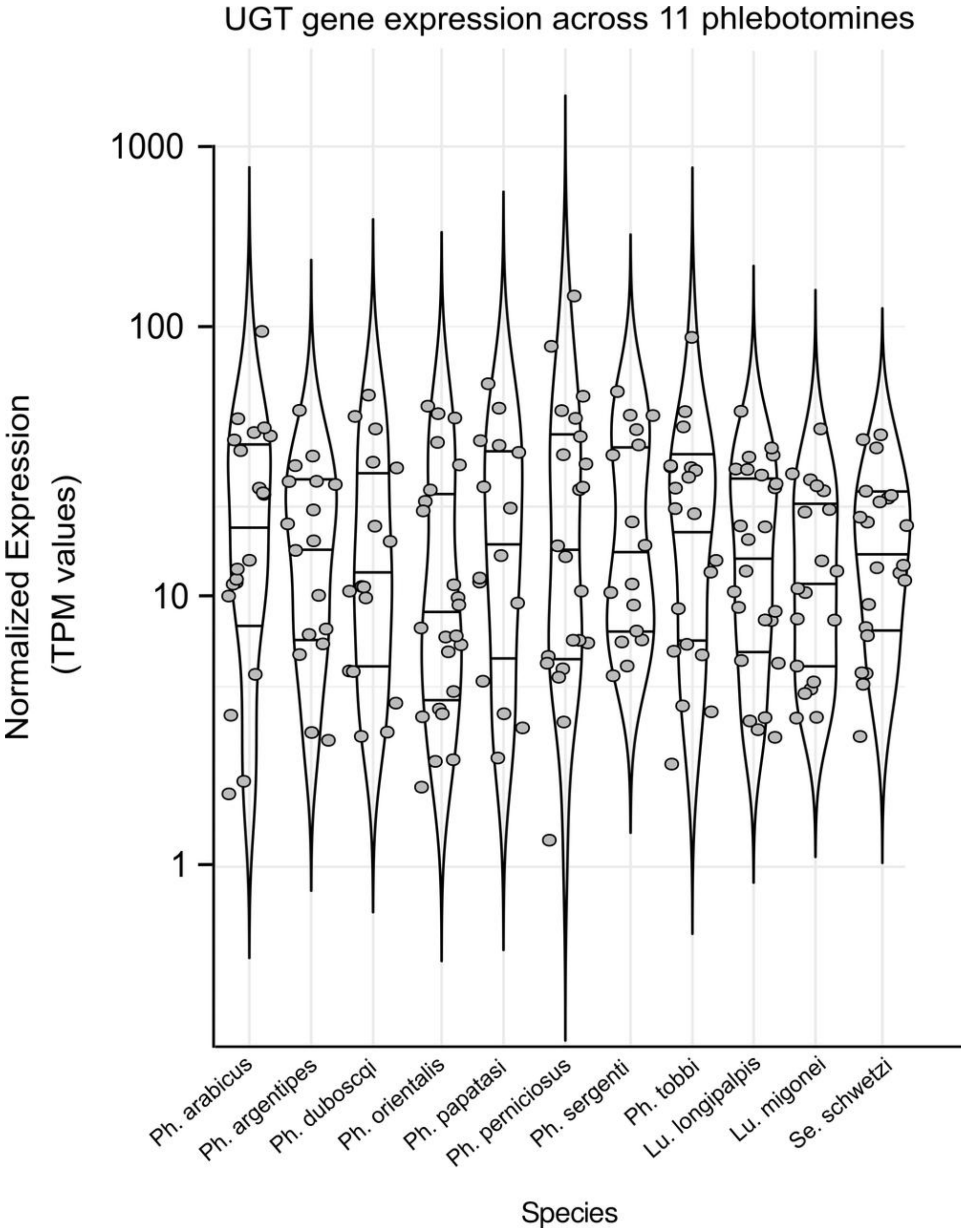

Supplementary Figure S13. Violin plot of UGT gene expression distribution across the 11 phlebotomines.

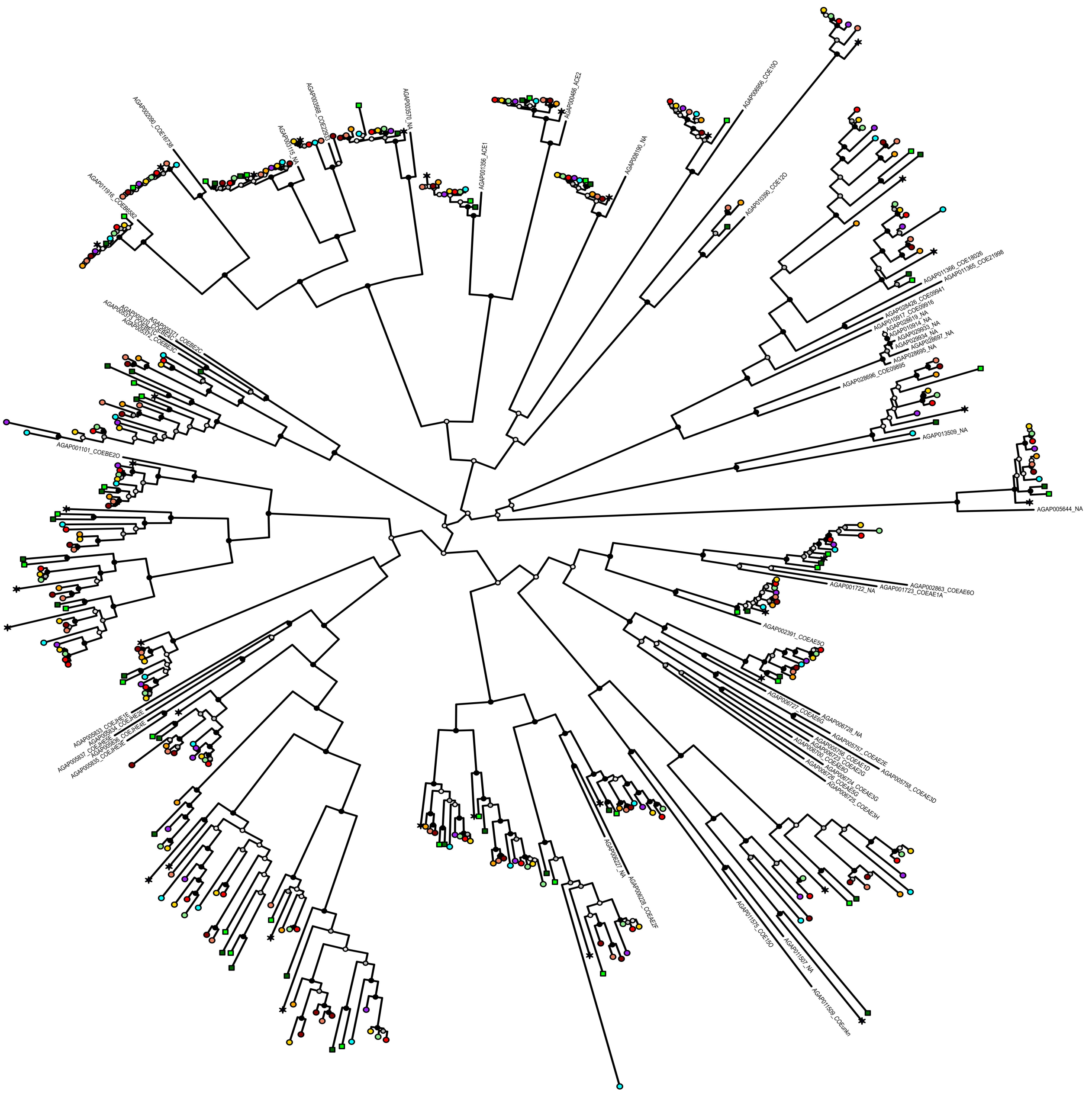

Supplementary Figure S14. Maximum likelihood phylogeny of 379 sand fly CCE genes with *A. gambiae* (n=52) as reference. Species are also denoted using color: *P. arabicus* (purple), *P. argentipes* (light cyan), *P. duboscqi* (brown), *P. orientalis* (gold), *P. papatasi* (dark red), *P. perniciosus* (turquiose), *P. sergenti* (orange), *P. tobbi* (red), *L. longipalpis* (dark green), *L. migonei* (light green) and *S. schwetzi* (blue). Bootstrap values are denoted with light grey (<50% bootstrap support), grey (50-75% bootstrap support) and black (>75% bootstrap support) circles.

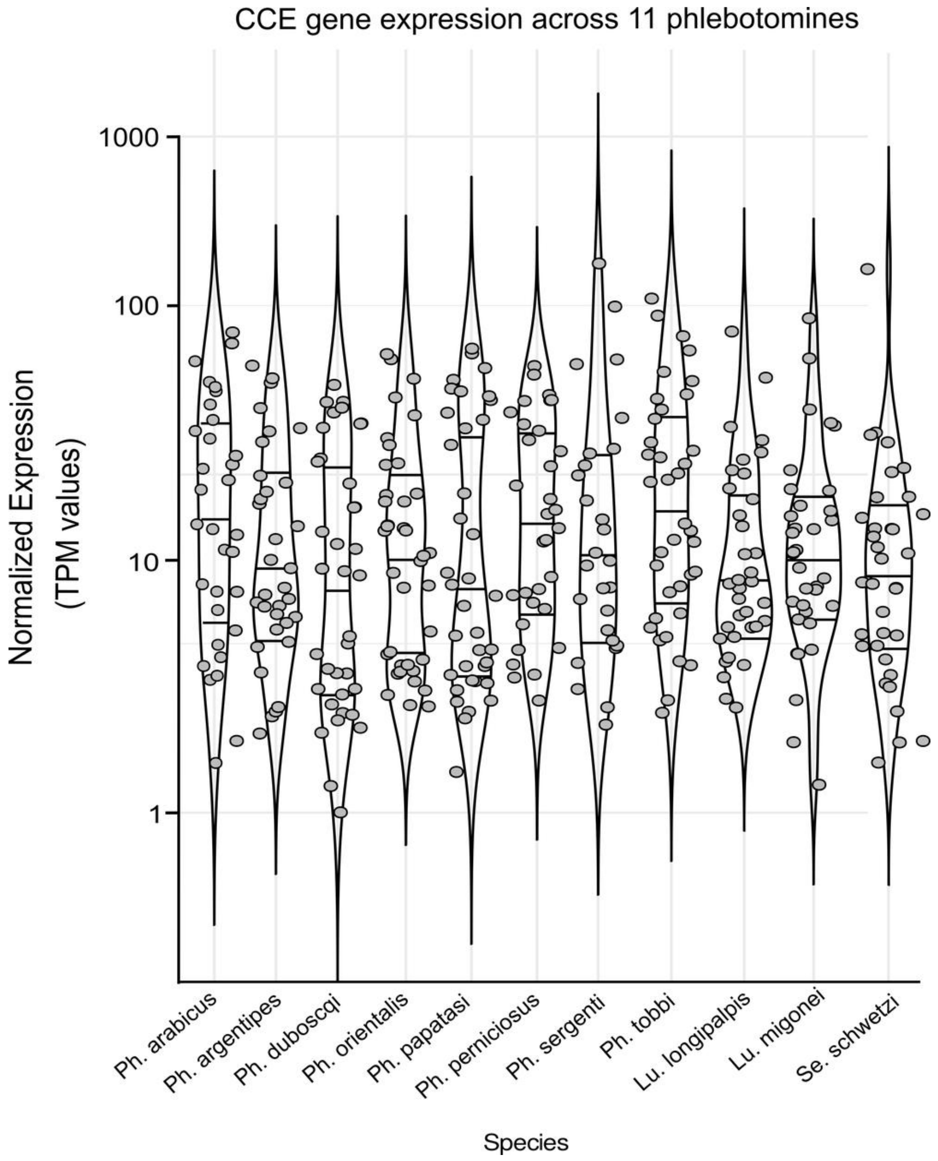

Supplementary Figure S15. Violin plot of CCE gene expression distribution across the 11 phlebotomines.

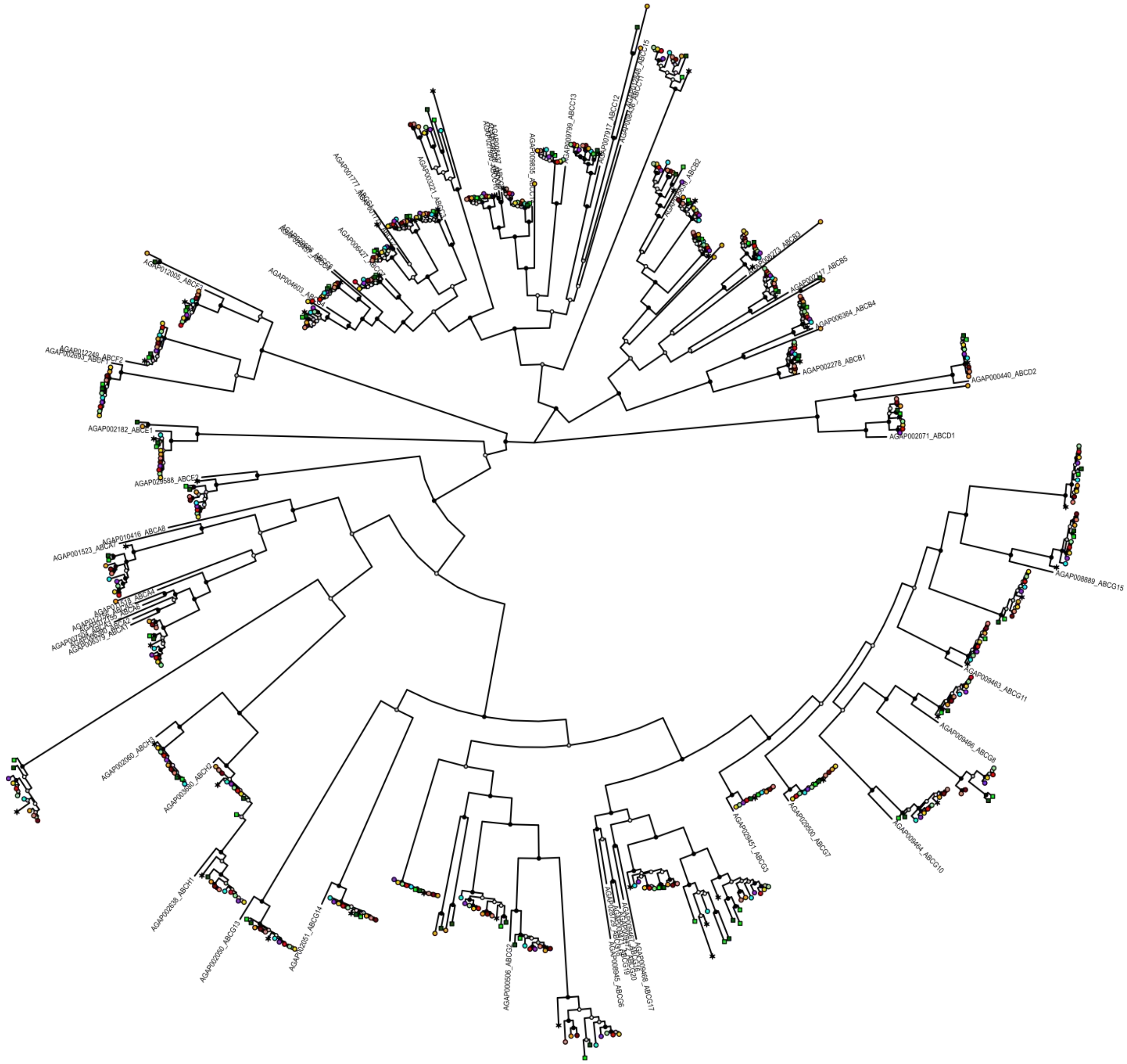

Supplementary Figure S16. Maximum likelihood phylogeny of 561 sand fly ABC transporter genes with *A. gambiae* (n=52) as reference. Sand fly species are denoted using a combination of color and shapes: *P. arabicus* (purple circle), *P. argentipes* (light cyan circle), *P. duboscqi* (brown circle), *P. orientalis* (gold circle), *P. papatasi* (dark red circle), *P. perniciosus* (turquiose circle), *P. sergenti* (orange circle), *P. tobbi* (red circle), *L. longipalpis* (green square), *L. migonei* (light green square) and *S. schwetzi* (blue star). Bootstrap values are denoted with light grey (<50% bootstrap support), grey (50-75% bootstrap support) and black (>75% bootstrap support) circles.

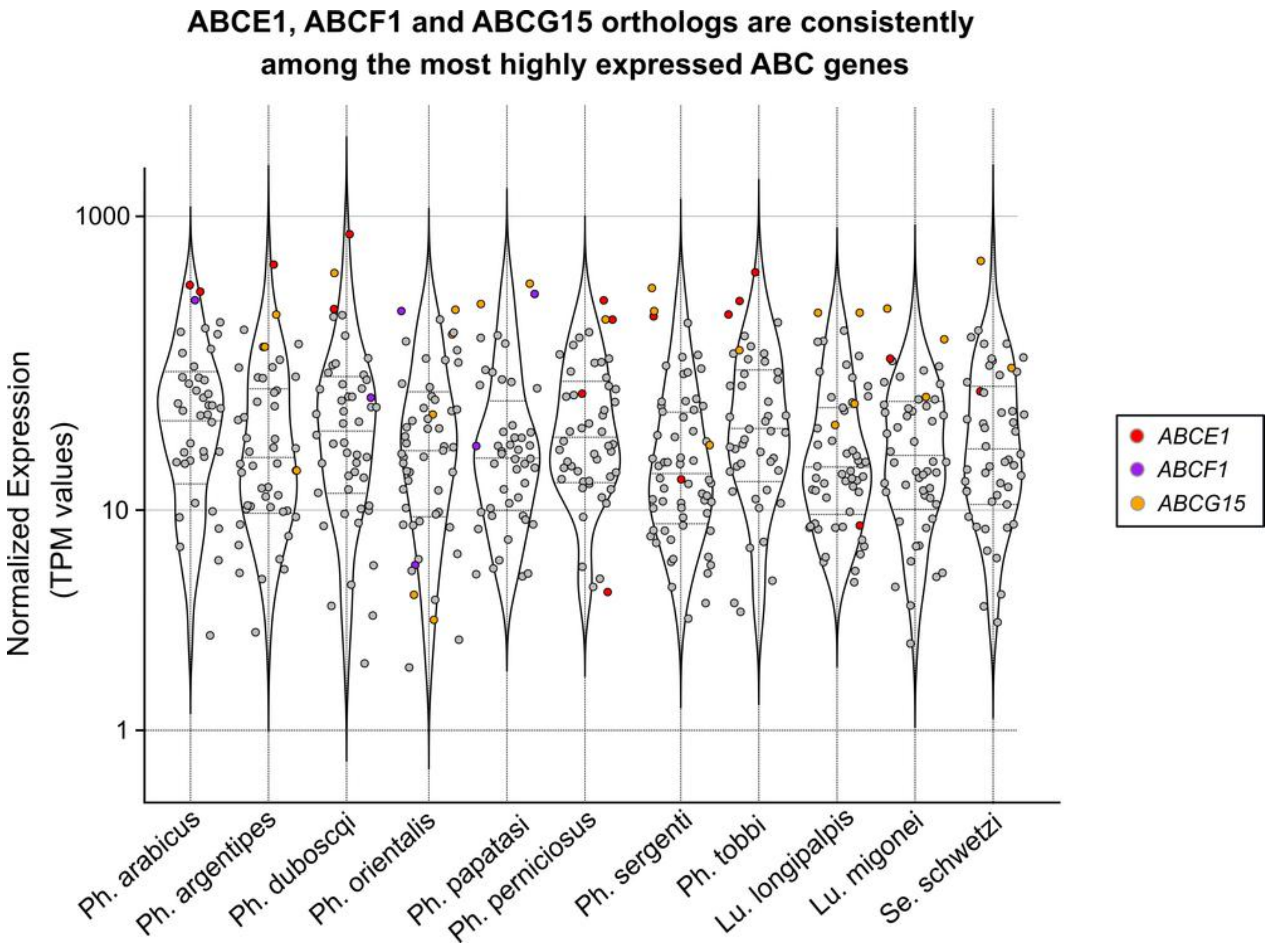

Supplementary Figure S17. Violin plot of ABC transporter gene expression distribution across the 11 phlebotomines. Orthologs of *ABCE1*, *ABCF1* and *ABCG15* are consistently among the most highly expressed ABC genes across the 11 phlebotomines.

Supplementary Table Legends S1-11

Supplementary Table S1. Number of reads per sample.

Supplementary Table S2. TransRate evaluation of Trinity and rnaSPAdes transcriptome assemblies.

Supplementary Table S3. BUSCO evaluation of Trinity and rnaSPAdes transcriptome assemblies.

Supplementary Table S4. BUSCO assessment of the 11 sand fly transcriptome assemblies and gene sets generated in this study. Reference genome assemblies of two sand fly and three mosquito vector species were also assessed for comparison.

Supplementary Table S5. Genomic resources used in this study.

Supplementary Table S6. Results of orthology analysis for the 11 sand fly and the 8 dipteran outgroup species.

Supplementary Table S7. Classification of CYP genes for each sand fly species.

Supplementary Table S8. Classification of GST genes for each sand fly species.

Supplementary Table S9. Classification of UGT genes for each sand fly species.

Supplementary Table S10. Classification of CCE genes for each sand fly species.

Supplementary Table S11. Classification of ABC transporter genes for each sand fly species.
